# A scoping review of Adverse, Benevolent, and Positive Childhood Experiences in military-connected children

**DOI:** 10.1371/journal.pmen.0000654

**Published:** 2026-07-22

**Authors:** Celia Mason, Faye Acton, Claire Camara, Paul Watson

**Affiliations:** 1 School of Nursing and Healthcare Sciences, Northumbria University, Newcastle Upon Tyne, United Kingdom; 2 Faculty of Health, Medicine and Social Care, Anglia Ruskin University, Cambridge, United Kingdom; Assiut University, EGYPT

## Abstract

Military-connected children and young people face unique stressors, such as relocations, educational disruptions, loss of friendships and parental deployments, which can contribute to Adverse Childhood Experiences (ACEs). However, Benevolent Childhood Experiences (BCEs) and Positive Childhood Experiences (PCEs) can act as protective factors. The military community offers stability, support, and strength, which may help buffer the effects of ACEs. Research into BCEs and PCEs for military-connected children is crucial to promoting positive outcomes and mitigating negative impacts over time. A scoping review was conducted to map literature regarding ACEs, BCEs and PCEs of Military Connected Children and Young People (MCCYP). Seven databases were searched (ASSIA, PsychARTICLES, PubMed, SAGE, Science Direct, Scopus and Web of Science), using key words and strategic search criteria. The Critical Skills Appraisal Program (CASP) tool was used for quality appraisal of the articles. Six papers met the inclusion criteria, of which five were USA based and one from the UK. All included articles focused on ACES, with one paper reporting PCEs in conjunction with ACEs. None were found regarding BCEs. Included articles contained research pertaining to current military connected children and the impact of ACEs on their current or future wellbeing. By undertaking the first scoping review explicitly examining ACEs, BCEs and PCEs among MCCYP, a significant gap in the evidence base was found.

## Introduction

Adverse Childhood Experiences (ACEs) refer to traumatic events which occur during childhood, from ten categories including abuse, neglect, and household dysfunction [1,2]. First introduced in a landmark study [1], ACEs have since been studied in multiple fields, such as education, public health, and medicine, due to their significant, long-term impacts on physical and mental well-being [3–5]. Having an empirically proven effect on outcomes, there now exists a substantial body of research on ACEs, highlighting their prevalence, impacts, and the mechanisms through which they influence developmental and health outcomes [6,7] Studies to date connect ACEs and various health outcomes, such as chronic diseases, mental health disorders, and behavioural challenges [7,8]. Identifying risk factors in early life may mitigate long term consequences of ACEs, improving health and wellbeing across the lifespan [9,10].

**Fig 1:**
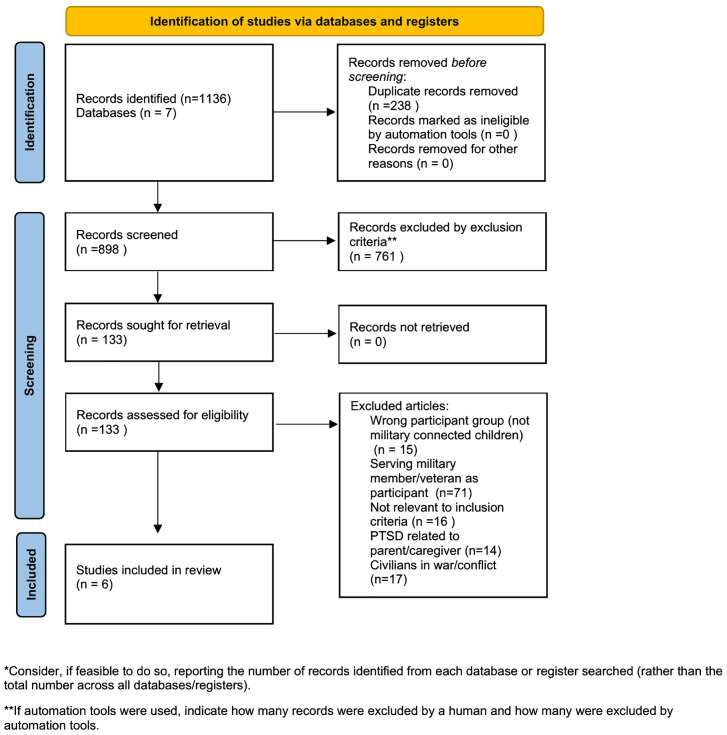
PRISMA flow chart depicting the screening process of the scoping review [13].

While Adverse Childhood Experiences (ACEs) have garnered extensive attention for their lasting negative impacts, research is increasingly highlighting the protective role of Benevolent Childhood Experiences (BCEs) and Positive Childhood Experiences (PCEs). BCEs, as foundational experiences of safety, care and relational stability, encompass nurturing relationships, opportunities for play, and supportive community environments, and contribute to resilience by fostering a sense of safety and belonging during formative years [11]. Similarly, PCEs, via positive experiences which promote growth, competence, and wellbeing, emphasise the importance of fostering emotional well-being through positive parental relationships, peer support, and opportunities for constructive coping mechanisms [12]. Less is currently known about these positive influences. Although often described collectively as counter-ACEs, BCEs and PCEs describe aspects of positive experiences in childhood, as shown in Fig 1. There are some overlapping themes within BCEs and PCEs, such as having adult support and feeling safe at home, however BCEs relate to early life conditions and PCEs refer to later emerging relational and psychosocial conditions. Therefore, inclusion of both concepts was important for to gain a comprehensive understanding for this review. Han et al., [14] found that although PCEs can buffer negative effects of ACEs, they have additional positive effects. It is thought that BCEs and PCEs function as counterbalancing forces, mitigating the harmful effects of childhood adversity, and promoting healthier developmental trajectories [15–17]. Table 1 depicts childhood experiences categorised within ACEs, BCEs and PCEs research.

**Table 1:** Examples of Adverse Childhood Experiences (ACEs), Benevolent Childhood Experiences (BCEs), and Positive Childhood Experiences (PCEs) identified in the literature.

Adverse Childhood Experiences (ACEs)	Benevolent Childhood Experiences (BCEs)	Positive Childhood Experiences (PCEs)
Physical abuse	At least one caregiver you felt safe with	Playtime
Sexual abuse	At least one good friend	Predictable, nurturing environments
Psychological abuse	Beliefs that comforted you	Recognition, praise, and acceptance
Physical neglect	Liked going to school	Support of other adult friends
Psychological neglect	At least one teacher who liked you	Feeling of belonging (school and community)
Witnessing domestic abuse	Good neighbours	Feeling safe and protected (by an adult at home)
Having a close family member who misused drugs or alcohol	Had an adult who supported or advised you	–
Having a close family member with mental health problems	Opportunities to have a good time	–
Having a close family member who served time in prison	Liked yourself or felt comfortable with yourself	–
Parental separation or divorce on account of relationship breakdown	Predictable home routine	–

Examples of ACEs adapted from Felitti et al., (1998) [1], BCEs were adapted from Narayan et al.’s (2018) [9] Benevolent Childhood Experiences Scale, and examples of PCEs were drawn from Crandall et al., (2019) [10] Not all categories were represented in the studies reviewed; cells marked with “—” indicate no examples were identified.

For Military Connected Children and Young People (MCCYP), the negative role of ACEs, and positive influence of BCEs and PCEs may be particularly pronounced [18]. Furthermore, military families represent a unique population in the context of ACEs research. Elements of military life, including frequent relocations, parental absence due to deployments, and living within an institution tied to conflict may elevate the risks of childhood adversity [19,20]. Strong familial bonds, supportive school environments, and access to mental health resources are critical in offsetting these unique stressors [21–23]. Often, service in the military is a ‘family business,’ with recruitment across multiple generations, having potential for recurring ACEs linked with Post Traumatic Stress Disorder (PTSD) within this [24,25].

The United Kingdom (UK) Ministry of Defence funded ‘Living in Our Shoes’ report [20] aimed to understand the needs of military families and assess support provision. This report highlights the importance of focusing on positive aspects of military family life, in addition to tackling negative aspects. Military life offers enhanced opportunities for BCEs and PCEs, despite its challenges. Factors such as personal resilience due to frequent relocations and parental deployments, the establishment of strong community ties within military families, and access to comprehensive formal support systems significantly contribute to positive outcomes [20,26,27]. Collectively, these elements increase access to BCEs and PCEs and provide protective mechanisms against ACEs.

There is a volume of retrospective ACEs research which aims to understand childhood experiences of adult serving military members and veterans, highlighting the relevance of ACEs experienced by this population [28–30]. Little is known to date regarding military connected children and their exposure to ACEs, BCEs and PCEs.

### Aims

This scoping literature review was undertaken to map existing literature and gain a deeper understanding of the current landscape of childhood experiences (ACEs, BCEs, and PCEs) in MCCYP. The review aimed to collect, synthesise, and analyse evidence regarding the application of the battery of screening tools, with a particular focus on how military-connected children self-report their own experiences of ACEs, BCEs, and PCEs.

## Methods

The scoping review utilised Arksey and O’Malley’s framework [31]. Scoping review was chosen as a method to map the broad area of childhood experience, including the nature and extent of evidence regarding ACEs, BCEs and PCEs [32]. The five stages of the review are as follows: 1. Identifying the research question; 2. Identifying relevant studies; 3. Study selection; 4. Charting the data and 5. Collating, summarising, and reporting the results.

To fulfil these requirements, seven databases were accessed on the 30/01/2025: ASSIA, PsychARTICLES, PubMed, SAGE, Science Direct, Scopus and Web of Science. To conduct the searches, search strings using key words were implemented as shown in Table 2. Key words were chosen to fulfil the study aims, capturing articles concerning current military-connected children with active serving members and adverse childhood experiences, positive childhood experiences, and/or benevolent childhood experiences. Inclusion criteria specified that the article must be in English, however the research could originate from any country. We did not specify publication year, as ACEs literature is expected to span from 1998 to present day [1]. Grey literature was not included, as the review aimed to capture the current landscape of published academic studies in an emerging field of literature. Additionally, literature regarding children of veterans or non active service members was excluded. The Preferred Reporting Items for Systematic reviews and Meta-Analyses (PRISMA) flow chart and check list was utilised to provide an overview of the study selection process (see S1 PRISMA Checklist) [33]. Table 2 contains the search strings used for each database.

**Table 2:** Search strings used for the scoping review.

*ASSIA*	*summary(Military) AND summary(Children) AND summary(“Adverse childhood experiences”) OR summary(“Benevolent childhood experiences”) OR summary(“Positive childhood experiences”)*
*PsychARTICLES*	*summary(Military) AND summary(Children) AND summary(“Adverse childhood experiences”) OR summary(“Benevolent childhood experiences”) OR summary(“Positive childhood experiences”)*
*PubMed*	*((((military[Title/Abstract]) AND (children[Title/Abstract])) AND (“Adverse childhood experiences”[Title/Abstract])) OR (“Benevolent childhood experiences”[Title/Abstract])) OR (“Positive childhood experiences”[Title/Abstract])*
*SAGE*	*military AND children AND “Adverse childhood experiences”* *military AND AND children AND “benevolent childhood experiences”* *military AND children AND “positive childhood experiences”*
*Science direct*	*military AND children AND “adverse childhood experiences” OR “ benevolent childhood experiences” OR “positive childhood experiences”*
*Scopus*	*military AND children AND “adverse childhood experiences” OR “ benevolent childhood experiences” OR “positive childhood experiences”*
*Web of Science*	*ALL=(military AND children AND “adverse childhood experiences” OR “benevolent childhood experiences” OR “positive childhood experiences”)*

Screening of title and abstract took place to identify relevant studies in the first round. This was followed by a full text reading of the article to ensure compatibility with the inclusion criteria. Articles which met the inclusion criteria were entered into a data charting spreadsheet, to capture relevant information for review by CM. Data charting took place using a structured, descriptive-analytical approach, ensuring consistency in recording key details (e.g., authors, publication year, study population, methodology, sample size and country).

The review was supported by PW. Inter reviewer and discrepancy agreement was reached regarding article inclusion via discussion between CM and PW, and CASP tool scoring. CASP cross-sectional study checklist [34] was used to assess the quality of the articles included in the scoping review. Quality appraisal via the CASP tool aimed to determine the relevance of the literature, and scoring took place to better understand the quality of the included material (see supporting information). Study quality did not influence inclusion or reporting of findings, as the number of articles was small.

## Results

Following the scoping review process, six articles met the criteria for inclusion. Fig 1 indicates the number of articles at each stage of the screening process.

Five articles originated from the USA [18,24,35–37] and one article was UK based [13]; however, it was funded by the United States (US) Department of Defence. Five articles used quantitative data analysis [18,24,35,37,13], and one article was a review relating to a US intervention for military-connected children [36]. The CASP appraisal indicated high methodological quality overall, reflecting clear sampling, valid measures, and robust analyses. In terms of the CASP tool, Crouch et al. [18], Bommersbach et al. [24], and Hinojosa et al. [37] met all 11 criteria. Clements-Nolle et al. [35] met 9, Fear et al. [13] 8, and Rossiter et al. [36] met 7 criteria, with lower scores reflecting limitations in generalisability and insufficient reporting of strategies to reduce bias. Nonetheless, the evidence base is narrow, and the research is largely US‑based, dependent on parent reports, and focused primarily on ACEs, with little attention to PCEs and no BCE‑focused work. This highlights clear gaps in who is represented and which constructs shape the current literature. Of the five articles which reported quantitative data, one article surveyed children [35], and four surveyed parents and caregivers [18,24,37,13]. In terms of large data sets, three articles used the National Survey of Children Health (NSCH) data, with Crouch et al. [18] reporting data from 2020/2021; Bommersbach et al., reporting data from 2018/2019 [24]; and Hinojosa et al., reporting data from 2017 to 2019 [37]. Clements-Knolle et al. analysed data from the 2017 Youth Risk Behaviour Survey (YRBS) [35]]; and Fear et al., collected data between 2010 and 2012 via postal questionnaire and telephone interview with fathers who served in Iraq and/or Afghanistan [13]. Questions around ACEs of physical, psychological, and sexual abuse are omitted from the NSCH due to the unlikelihood of this being reported accurately by parents and caregivers, and fear of people not completing the questionnaire due to these questions. The YRBS contains six ACEs based questions about sexual, physical, and verbal abuse, domestic violence, mental illness, and substance use in the family home. As with any self-report measure, this may be subject to bias or misrepresentation [35].

Crouch et al. [18]. examined PCEs in conjunction with ACEs [15], however, no literature relating to military-connected children was found around BCEs. Article publication dates ranged from 2018 to 2024. Table 3 contains the six accepted articles for this scoping review, with a summary of article characteristics.

**Table 3:** Summary of characteristics of articles for inclusion in the scoping review.

Study	Country	Design/ Data Source	Sample Characteristics	Measures (ACEs/ PCEs)	CASP Score	Key Findings
Crouch et al., 2024 [18]	USA	Quantitative; NSCH 2020–2021	Parents/caregivers of military-connected children	ACEs (excluding abuse items); PCEs (supportive relationships, community engagement)	11/11	Military families show higher dysfunction vs civilians; PCEs buffer negative outcomes, improving emotional/social wellbeing.
Bommersbach et al., 2022 [24]	USA	Quantitative; NSCH 2018–2019	Parents/caregivers of military-connected children	ACEs (NSCH items); family outcomes	11/11	Military families had higher income, less divorce, better healthcare access; but increased ADHD/autism diagnoses in children.
Clements-Nolle et al., 2021 [35]	USA	Quantitative; YRBS 2017	Adolescents in military families	ACEs (6 items: abuse, DV, mental illness, substance use); suicidality	9/11	Military adolescents had higher suicide attempts; ACEs mediated link between parental military service and suicidality.
Rossiter et al., 2020 [36]	USA	Review of US intervention programs	Military-connected children (secondary trauma focus)	Toxic stress, counter-ACEs (proposed interventions)	7/11	Highlighted toxic stress from parental service; recommended school-based, family-centred, and mental health interventions.
Hinojosa et al., 2024 [37]	USA	Quantitative; NSCH 2017–2019	Parents/caregivers in military, veteran, and civilian families	ACEs (NSCH items)	11/11	Military/veteran families disproportionately affected by substance use, divorce, family violence; PTSD/deployment stress key drivers.
Fear et al., 2018 [13]	UK (US DoD funded)	Quantitative; postal survey + interviews (2010–2012)	Fathers deployed to Iraq/Afghanistan; children indirectly assessed	ACEs via parental PTSD impact	8/11	Children’s trauma linked to paternal PTSD; deployment stress transmitted intergenerationally.

Across the literature, a consistent theme emerges regarding the differential exposure to ACEs and family dysfunction in military families compared with civilian families, with four studies [18,24,35,37] reporting elevated risk. Clements-Nolle et al. [35] investigated attempted suicide among adolescents in military families, finding that ACEs, such as parental mental health and family instability due to deployment, mediate the relationship between family military service and suicidality. Similarly, Hinojosa et al. [37] compared ACEs in military, veteran, and civilian families, finding that military and veteran families experience some ACEs in proportion to civilian families, such as financial worries, living with someone with mental ill health, experiencing violence and racial discrimination; however veteran and military families are disproportionately affected by higher rates of substance use, divorce and family violence in comparison to civilian families, particularly when parental combat related PTSD and deployment-related stress are factors. These findings align with earlier findings by Fear et al. [13], who explored the effects on military children of paternal deployment to Iraq and Afghanistan and the development of PTSD in military fathers. Their research found mechanisms of stress transmission linked to paternal PTSD. Together, the studies illustrate a transgenerational pattern of risk, in which parental military factors and experience can influence children’s developmental environments.

Bommersbach et al. [24] examine transgenerational factors associated with military service, comparing the experiences of children of veterans with those of children of nonveterans, finding positive elements such as increased income, less divorce and separation between parents and better access to medical care in military families. Interestingly, they also found an increase in clinically recognised behavioural conditions in military-connected children such as ADHD and autism. This study took place in the US; therefore, this finding may be impacted by military families having greater access to healthcare insurance. Linking this to Hinojosa et al., [37] findings, the transgenerational perspective underscores the importance of addressing the cumulative effects of military service across family generations to promote better outcomes for children.

A parallel theme across the literature concerns the role of protective and promotive experiences (PCEs) in mitigating the impact of ACEs. Crouch et al. [18] examined the presence of both ACEs and PCEs among U.S. military-connected children, noting that those who report higher levels of PCEs (Fig 1), such as supportive relationships and community engagement, tend to fare better emotionally and socially. Rossiter et al. [36] complemented this finding with an exploration of the concept of toxic stress in military-connected children, emphasising secondary trauma associated with parental military service post-9/11, Although they do not explicitly label their recommended supports as PCEs, their proposed interventions, such as early identification of ACEs, targeted support for parents with PTSD, school‑based programmes, and family‑centred services function as counter‑ACEs, directly aligning with Crouch et al.’s findings. This is in keeping with findings by Fear et al., [13]. Rossiter et al., [36] propose interventions which aim to reduce toxic stress experienced by military-connected children, such as access to mental health services, school-based support programs, and family-centred interventions. Furthermore, Rossiter et al., [36] posit that early identification of ACES within military families and targeted interventions for parents with PTSD can improve long term outcomes. This supports findings by Crouch et al. [18], who highlight that military-connected children with greater access to PCEs such as supportive relationships and community engagement have better outcomes.

Collectively, these studies reveal a heterogeneous landscape. Military‑connected children face elevated ACE exposure driven by deployment‑related instability and parental PTSD yet may also benefit from structural advantages and access to PCEs that buffer risk. This synthesis underscores the importance of adopting a nuanced, multi‑level approach that recognises both the vulnerabilities and strengths within military families and highlights the need for interventions that simultaneously reduce ACEs and cultivate PCEs to promote long‑term wellbeing.

## Discussion

This scoping review identified a limited body of research examining ACEs, BCEs and PCEs among military‑connected children and young people (MCCYP). Across the available studies, children’s perspectives were largely absent, with most data reported by parents or caregivers. Only one study directly surveyed young people [35]. Furthermore, none of the included studies used dedicated ACEs, BCEs or PCEs screening tools; instead, ACE‑related items were embedded within broader questionnaires.

In recent years, literature regarding ACEs in military populations has increased, however the majority of this refers to ACEs from a retrospective adult perspective. Little is known about ACEs, BCEs, and PCEs in relation to MCCYP, specifically and importantly from their perspective. MCCYP are a population who encounter unique, additional stressors in comparison to children from civilian families. They can experience stressors such as frequent relocations, parental deployment, and reintegration challenges, which may contribute to ACEs. However, military life also offers opportunities for BCEs and PCEs, including formal and informal support systems encouraging resilience, coping skills and social connection [38,39]. Given this complexity, it is essential not only to conduct research with and for MCCYP, but to embed participatory approaches that ensure their influence, and enable power sharing and co‑ownership of research processes [40]. Development of policy and practice around participatory inclusion of MCCYP is imperative, thus providing value-based interventions that meet the needs of this population [40].

### Significance and implications of the identified gap

A clear research gap has been identified during this scoping review. Military- connected children and young people are a substantial population facing well documented unique stressors [16,17], yet policies, service provision and interventions are being designed without empirical evidence specific to this population from their perspective. As it stands, current service provision for this population of children and young people are based on retrospective research (relying on memory) or research from the perspective of parents/ carers and adult service providers conducting ACE reporting, which does not capture the developmental, relational, and contextual realities of what it is like to be a military connected child or young person today from a child’s perspective.

This gap in knowledge is due to methodological challenges inherit with researching children and young people (Ethical difficulties) and particularly researching military populations (access restrictions, mobility etc) and the interdisciplinary of the topic, which falls between child development, family studies and research on military health and wellbeing. However, the issue had become increasingly urgent given the uncertainty of global security and a growing recognition of intergenerational trauma [31,34].

### Unique contributions of this review

This scoping review provides the first review mapping specifically examining ACEs, BCEs and PCEs of MCCYP, revealing two critical findings that advance understanding in this field:

1. The absence of the child/young persons voice: Only one study [30] directly surveyed children, demonstrating a methodological bias towards adult reporting, which misses the lived experience of that child or young person.
2. The lack of BCEs Evidence: Despite the theoretical importance of surveying BCEs none of the studies within this review examined the benevolent experience of MCCYP. The lack of examining represents a missed opportunity to understand the protective and positive aspects of a child’s life.

### Future research methodological recommendations

By prioritising co-designed translational research regarding BCEs and PCEs alongside ACEs with MCCYP, interventions can be designed to strengthen protective factors, enhance resilience, and optimise long-term outcomes for children in military families. Given the evidence around specific stressors experienced by military-connected children [18,19], screening children and young people from military connected families for ACEs, BCEs and PCEs designed by and for them offers opportunity for an increased uptake in health related screening. This provides an opportunity for early intervention to assess, formulate and action any concerns relating to the ecosystem of that specific child, for example, the interconnected influences of their health and wellbeing, their connections with family and friends, their educational attainment and access to community service provision. [41].

Based on our review, we propose the following methodological recommendations:

1. Develop military-adapted screening tools: Existing screening tools to measure ACEs, BCEs and PCEs could be adapted to incorporate military specific experiences.
2. Co-Production with MCCYP: Future research should include children and young people to be more than just participants, but be a valued part of the design, and validation of adapted or newly developed screening tools.
3. Longitudinal and comparative Designs: Undertake longitudinal research to follow and measure the temporal relationships between experiences within military life and outcomes. In addition, include military and civilian comparisons to identify specific risks and protective factors.

### Theoretical contribution

This review synthesizes existing evidence to propose a Military Connected Childhood Experiences Framework that recognises:

1. Military life creates unique ACEs (deployment or combat related trauma (physical or psychological), parental loss due to deployments etc)
2. Military culture simultaneously provides distinct opportunities for BCEs/PCEs (structured support, community identity, access to resources).
3. The balance between these experiences is dynamic and influenced by factors such as deployment frequency, parental mental health, and community support quality.

This framework moves beyond deficit-based models to recognise both adversities and assets in military-connected childhoods, providing a foundation for strength-based intervention design. In practical terms, the framework may inform the development of screening approaches that capture both military-specific adversities and protective experiences, rather than relying solely on cumulative ACE counts. It may also guide intervention design by encouraging services to simultaneously mitigate identified risks while strengthening protective systems such as peer networks, school belonging, and family resilience. At a policy level, the framework offers a structured lens through which military-connected childhood can be situated within broader child health inequalities discourse, supporting proportionate and context-sensitive commissioning approaches. Early operationalisation of these principles within trauma-informed service models has been illustrated through the Identify–Connect–Engage (ICE) framework [41] which demonstrates how conceptual domains can translate into practice-oriented assessment and engagement strategies.

### Strengths and limitations

In consideration of strengths of this scoping review, it was carried out in an under researched area of literature, to understand the current landscape and offer insights into future research directions of importance. Findings have highlighted research gaps in regard to current use of ACEs, BCEs and PCEs screening and tools with military-connected children; and the lack of the child’s voice in both the screening of military-connected children and within the co-design of the research. The aim of a scoping review is to offer a broad yet structured map of current, relevant literature. Use of Arksey and O’Malley’s framework [28] to conduct this scoping review offered an analytic framework for the review, without being prescriptive regarding synthesis of the literature. Additionally, a strength of this review is the use of the CASP tool, to understand the rigour of the articles found following the screening process.

In terms of limitations, we acknowledge that only six studies met the inclusion criteria, and none directly examined BCEs. While the evidence base was small, we used Arksey and O’Malley’s scoping review framework [31] because the primary aim was not to synthesise findings in depth but rather to map the extent, nature, and characteristics of available research on ACEs, BCEs, and PCEs in military-connected children. A scoping review is particularly valuable in emerging or under-researched fields, as it allows for the systematic identification of evidence gaps, clarification of concepts, and guidance for future research priorities. In this case, the limited number of eligible studies is itself a key finding, underscoring the paucity of empirical research and highlighting the need for more focused investigations into BCEs and PCEs. A further limitation is the strong geographic concentration of existing research, which is dominated by studies from the United States. This lack of international representation reduces cultural diversity within the evidence base and constrains the applicability of findings to MCCYP in other military systems and sociocultural contexts.

Additionally, utilisation of ACEs questionnaires differed across the studies. The NSCH included questions around seven of the ten expected ACEs factors, excluding questions about abuse due to fears this would hinder data collection [37]. It is disappointing not to find articles around BCEs and only one article regarding PCEs, however this offers encouragement to researchers in this field. We acknowledge that excluding grey literature may mean some relevant reports or policy documents were not captured. Reports which have been produced by charities, government or military policy papers, and material produced by MCCYP family support organisations may have captured insights which have not been found within this review. Equally, literature which did not specifically use the terminology chosen to construct the search strings, but may describe an element of ACEs, BCEs or PCEs would have been excluded from inclusion. However, this review was intended to map the peer-reviewed academic evidence base, as a necessary first step towards understanding the current literature in this field. Additionally, to understand the availability of literature which does not specifically mention ACEs, BCEs or PCEs, but discusses themes which would be relevant to these, a larger study would be required.

The gaps identified in this review have clear implications for the systems supporting MCCYP. For clinical practice, the absence of child‑reported data and the lack of military‑adapted screening tools highlight the need for developmentally appropriate, trauma‑informed assessment that captures both adversity and protective experiences. Within education, schools are well placed to recognise stressors linked to mobility and deployment, and to strengthen promotive experiences such as belonging, stability and supportive relationships. Military family services similarly require coordinated, preventative approaches that integrate ACE/BCE/PCE screening into routine support and respond to the influence of parental mental health, particularly deployment‑related PTSD. Ensuring that children’s perspectives inform service design is essential for developing interventions that reflect their lived realities and enhance resilience across clinical, educational, and military contexts.

## Conclusion

In conclusion, this scoping review highlights a significant gap in the literature on the exposure of military-connected children to ACEs, BCEs, and PCEs. Existing research is limited in scope, predominantly caregiver-reported and having little inclusion of the child’s voice. Additionally, the majority of research within this field has taken place in the US, reflecting US-based military systems and datasets. Whilst this review demonstrates there is a lack of research surround the voice of MCCYP, it also demonstrates the need for specific research to be carried out with and for MCCYP. By that we mean researchers need to move past researching on MCCYP and move to participatory with MCCYP. As the findings within this paper highlight, the lens of a child or young person are missing from the development of screening tools and research itself, resulting in a significantly different lens in which research is performed. This is seen to be research conducted through an adult, and specifically an adult researcher’s lens, which results in tools and their implementation to policy and practice being designed and implemented from an adults perspective. This point is made clearer by the lack of child or young person specific self-reporting screening tools for ACEs, BCEs, or PCEs. While there is growing literature on ACEs in military populations, it mostly focuses on retrospective adult experiences, with limited exploration of how these factors affect military-connected children directly. Therefore, we argue MCCYP need to be part of the whole research process, from its design to its implementation. By including the child or young person in the whole research process we argue any future research on health-related screening should focus on both protective factors (BCEs and PCEs) alongside adverse experiences (ACEs) to provide a balanced perspective which inform interventions that strengthen the emotional health and well-being of this population and improve outcomes for military-connected children.

Looking ahead, there is a clear need for the development of balanced screening approaches that assess protective and promotive experiences in the context of MCCYP experiences of adversity. Tools should be co-produced with MCCYP and tailored to military life experience. Future screening models can better inform interventions which enhance wellbeing, resilience and long-term outcomes for MCCYP.

## Supporting information

S1 PRISMA Checklist Preferred Reporting Items for Systematic reviews and Meta-Analyses extension for Scoping Reviews (PRISMA-ScR). (DOCX)

S1 Text Critical Appraisal Skills Programme (CASP) for descriptive/cross sectional studies reporting of Clements-Nolle et al., (2021). (DOCX)

S2 Text Critical Appraisal Skills Programme (CASP) for descriptive/cross sectional studies reporting of Crouch et al., (2024). (DOCX)

S3 Text Critical Appraisal Skills Programme (CASP) for descriptive/cross sectional studies reporting of Rossiter et al., (2020). (DOCX)

S4 Text Critical Appraisal Skills Programme (CASP) for descriptive/cross sectional studies reporting of Bommersbach et al., (2022). (DOCX)

S5 Text Critical Appraisal Skills Programme (CASP) for descriptive/cross sectional studies reporting of Fear et al., (2018). (DOCX)

S6 Text Critical Appraisal Skills Programme (CASP) for descriptive/cross sectional studies reporting of Hinojosa et al., (2024). (DOCX)
