# Supplementary material for: A scoping review of Adverse, Benevolent, and Positive Childhood Experiences in military-connected children
Source: PLOS Ment Health. 2026 Jul 22;3(7):e0000654. doi: 10.1371/journal.pmen.0000654 (PMC13390850; doi:10.1371/journal.pmen.0000654)
Supplement: S5 Text — (DOCX) [file pmen.0000654.s006.docx]

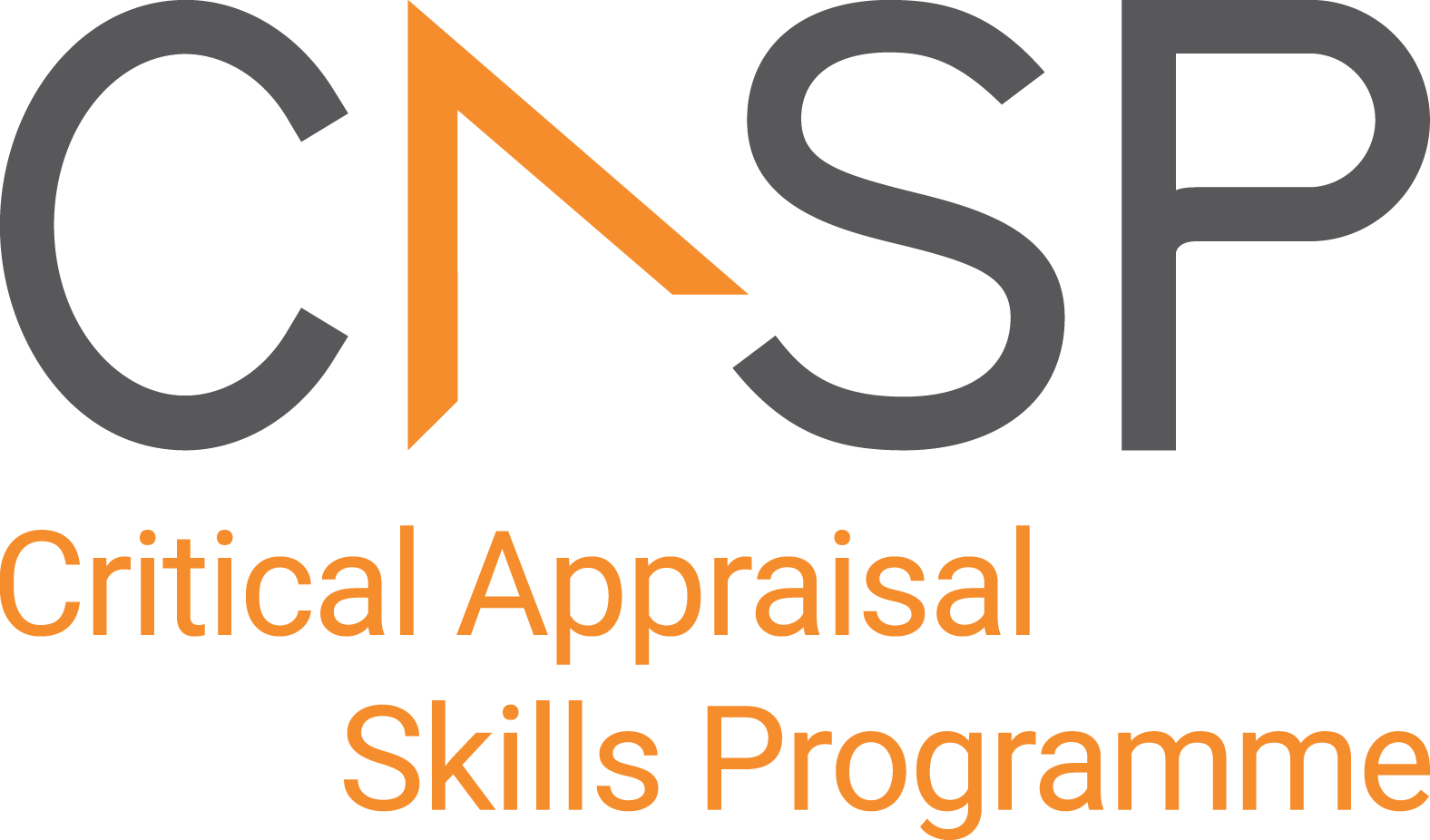
CASP Checklist:

For Descriptive/Cross-Sectional Studies

| **Reviewer Name:** |  |
| --- | --- |
| **Paper Title:** | Impact of paternal deployment to the conflicts in Iraq and Afghanistan and paternal post-traumatic stress disorder on the children of military fathers |
| **Author:** | Nicola T. Fear, Ruth V. Reed, Sarah Rowe, Howard Burdett, David Pernet, Alyson Mahar, Amy C. Iversen, Paul Ramchandani, Alan Stein* and Simon Wessely* |
| **Web Link:** | doi: 10.1192/bjp.2017.16 |
| **Appraisal Date:** | 03/04/2025 |

During critical appraisal, never make assumptions about what the researchers have done. If it is not possible to tell, use the “Can’t tell” response box. If you can’t tell, at best it means the researchers have not been explicit or transparent, but at worst it could mean the researchers have not undertaken a particular task or process. Once you’ve finished the critical appraisal, if there are a large number of “Can’t tell” responses, consider whether the findings of the study are trustworthy and interpret the results with caution.

| **Section A: Are the results valid?** | | |
| --- | --- | --- |
| 1. Did the study address a clearly focused issue? | Yes  No  Can’t Tell | |
| *CONSIDER:*  *A question can be ‘focused’ in terms of*   - *the population studied* - *the risk factors studied* - *is it clear whether the study tried to detect a beneficial or harmful effect* - *the outcomes considered* | | |
| 1. Did the authors use an appropriate method   to answer their question? | Yes  No  Can’t Tell | |
| *CONSIDER:*   - *Is a descriptive/cross-sectional study an appropriate way of answering the question* - *did it address the study question* | | |
| 1. Were the subjects recruited in an acceptable way? | Yes  No  Can’t Tell | |
| *CONSIDER:*  *We are looking for selection bias which might compromise the generalisability of the findings:*   - *Was the sample representative of a defined population* - *Was everybody included who should have been included* | | |
| 1. Were the measures accurately measured to reduce bias? | Yes  No  Can’t Tell | |
| *CONSIDER:*  *Look for measurement or classification bias:*   - *did they use subjective or objective measurements* - *do the measurements truly reflect what you want them to (have they been validated)* | | |
| 1. Were the data collected in a way that addressed the research issue? | Yes  No  Can’t Tell | |
| *CONSIDER:*   - *if the setting for data collection was justified* - *if it is clear how data were collected (e.g., interview, questionnaire, chart review)* - *if the researcher has justified the methods chosen* - *if the researcher has made the methods explicit (e.g. for interview method, is there an indication of how interviews were conducted?)* | | |
| 1. Did the study have enough participants to minimise the play of chance? | | Yes  No  Can’t Tell |
| *CONSIDER:*   - *if the result is precise enough to make a decision* - *if there is a power calculation. This will estimate how many subjects are needed to produce a*   *reliable estimate of the measure(s) of interest.* | | |
| 1. How are the results presented and what is the main result? | Yes  No  Can’t Tell  **Did not associate paternal deployment with ACEs in children** | |
| *CONSIDER:*   - *if, for example, the results are presented as a proportion of people experiencing an outcome, such as risks, or as a measurement, such as mean or median differences, or as survival curves and hazards* - *how large this size of result is and how meaningful it is* - *how you would sum up the bottom-line result of the trial in one sentence* | | |
| 1. Was the data analysis sufficiently rigorous? | Yes  No  Can’t Tell | |
| *CONSIDER:*   - *if there is an in-depth description of the analysis process* - *if sufficient data are presented to support the findings* | | |
| 1. Is there a clear statement of findings? | Yes  No  Can’t Tell | |
| *CONSIDER:*   - *if the findings are explicit* - *if there is adequate discussion of the evidence both for and against the researchers’ arguments* - *if the researchers have discussed the credibility of their findings* - *if the findings are discussed in relation to the original research questions* | | |
| 1. Can the results be applied to the local population? | Yes  No  Can’t Tell | |
| *CONSIDER:*   - *the subjects covered in the study could be sufficiently different from your population to cause concern.* - *your local setting is likely to differ much from that of the study* | | |
| 1. How valuable is the research? | | Yes  No  Can’t Tell |
| *CONSIDER:*   - *one descriptive/cross-sectional study rarely provides sufficiently robust evidence to recommend changes to clinical practice or within health policy decision making* - *if the researcher discusses the contribution the study makes to existing knowledge (e.g., do they consider the findings in relation to current practice or policy, or relevant research-based literature?)* - *if the researchers have discussed whether or how the findings can be transferred to other populations* | | |

| **APPRAISAL SUMMARY**: *List key points from your critical appraisal that need to be considered when assessing the validity of the results and their usefulness in decision-making.* | | |
| --- | --- | --- |
| **Positive/Methodologically sound** | **Negative/Relatively poor methodology** | **Unknowns** |
| **Methodologically sound**  **Adds to knowledge** | **Parental self report by mothers, in regard to father/child relationship** |  |

**Referencing recommendation:**

CASP recommends using the Harvard style referencing, which is an author/date method. Sources are cited within the body of your assignment by giving the name of the author(s) followed by the date of publication. All other details about the publication are given in the list of references or bibliography at the end.

Example:

*Critical Appraisal Skills Programme (2024). CASP (insert name of checklist i.e. cross sectional Checklist.) [online] Available at: insert URL. Accessed: insert date accessed.*

**Creative Commons**

©CASP this work is licensed under the Creative Commons Attribution – Non-Commercial- Share A like. To view a copy of this licence, visit <https://creativecommons.org/licenses/by-nc-sa/4.0/>

**
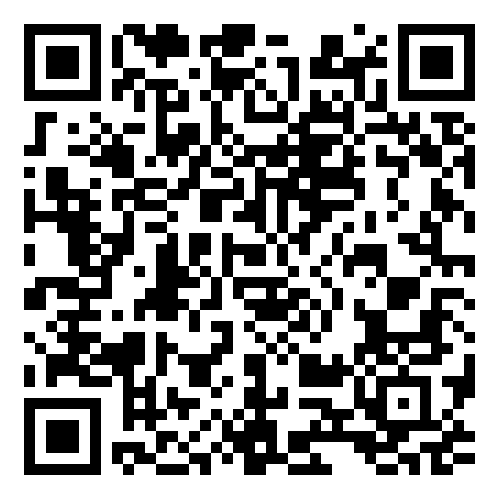
Need further training on evidence-based decision making?** Our online training courses are helpful for healthcare educational researchers and any other learners who:

- Need to critically appraise and stay abreast of the healthcare research literature as part of their clinical duties.
- Are considering carrying out research & developing their own research projects.
- Make decisions in their role, whether that be policy making or patient facing.

**Benefits of CASP Training:**

- Affordable – courses start from as little as £6
- Professional training – leading experts in critical appraisal training
- Self-directed study – complete each course in your own time
- 12 months access – revisit areas you aren’t sure of and revise
- CPD certification - after each completed module

Scan the QR code below or visit <https://casp-uk.net/critical-appraisal-online-training-courses/> for more information and to start learning more.
