# Supplementary material for: A scoping review of Adverse, Benevolent, and Positive Childhood Experiences in military-connected children
Source: PLOS Ment Health. 2026 Jul 22;3(7):e0000654. doi: 10.1371/journal.pmen.0000654 (PMC13390850; doi:10.1371/journal.pmen.0000654)
Supplement: Attachment — Submitted filename: Comment table V3 Jan 26.docx [file pmen.0000654.s011.docx]

| Reviewer | Comment | Response |
| --- | --- | --- |
| 1 | It seems as if the authors have addressed the concerns of other reviewers. I, again, stand by my original assertion that if this meets the criteria for publishing from the journal, I am in support of publishing it. My original concern had nothing to do with the writing/analysis of the authors, but rather a concern or question about the overall application of the content. I am in support of the editor's decision. | Thank you for your response to the edited manuscript and the time taken to support this towards publication. |
| 2 | Given the very small number of included studies (n = 6), the Results section occasionally reads more like an extended summary of individual papers. Consider synthesising findings more explicitly around themes (e.g., measurement gaps, informant bias, intergenerational trauma). | We have edited the results section, to condense and synthesise findings more explicitly. Themes have also been noted throughout this section. |
| 2 | The discussion section is relatively long and could benefit from tighter structuring with clearer subheadings or more concise synthesis in some sections. | We have removed and condensed some of the text from the discussion section as suggested. Thank you for bringing this to our attention. |
| 2 | Some arguments (e.g., the urgency of participatory research, limitations of adult-reported data) are repeated across multiple subsections and could be streamlined. | Thank you for drawing this to our attention. While condensing the results and discussion sections we have removed repeated references to these areas. |
| 2 | While strengths and limitations are addressed thoroughly, the implications for clinical practice, education systems, and military family services could be more explicitly articulated. | Thank you for this suggestion. We have added a brief section outlining implications in these areas, titled “Implications for Clinical Practice, Education, and Military Family Services”. |
| 2 | This manuscript addresses an important and under-researched area and makes a meaningful contribution by mapping the near-absence of BCEs and PCEs research and especially child-reported data among military-connected children. With some tightening of the introduction and discussion, clearer articulation of research questions, and stronger synthesis in the Results section, the paper would be well positioned for publication in a mental health journal with an applied and policy-oriented readership. | Thank you for your comments and guidance. We hope that the amendments we have made strengthen this manuscript towards publication of this paper in an under researched field |
