# Supplementary material for: A scoping review of Adverse, Benevolent, and Positive Childhood Experiences in military-connected children
Source: PLOS Ment Health. 2026 Jul 22;3(7):e0000654. doi: 10.1371/journal.pmen.0000654 (PMC13390850; doi:10.1371/journal.pmen.0000654)
Supplement: Attachment — Submitted filename: ACEs comment table October 25.docx [file pmen.0000654.s009.docx]

| Reviewer | Comment | Response |
| --- | --- | --- |
| 1 | While I appreciate the concepts presented in the manuscript, the conclusions do not extend past the idea that there is a lack of literature regarding the topic. While I have no doubt that this is the case, it is not just additional research that is required, but rather research period is required to understand this. | Thank you for your review of this manuscript. We have expanded on research needs and methods in the discussion section. |
| 2 | Conceptual Clarity (ACEs, BCEs, PCEs): The manuscript provides useful definitions of ACEs, BCEs, and PCEs, but at times these categories are blurred. For instance, the distinction between BCEs and PCEs is not consistently sustained throughout the paper. Clarifying how these differ and why both are included (rather than subsumed as “counter-ACEs”) would enhance theoretical coherence. | Thank you for this recommendation. We have added text to differentiate between BCEs and PCEs in relation to ACEs. Please see page 4 line 86, and page 3, line 78/79 |
| 2 | Scope and Aims of the Review: The stated aim is to “map existing literature and gain a deeper understanding of the current landscape of ACEs, BCEs, and PCEs in military-connected children.” However, only six studies were identified, and none directly addressed BCEs. This raises the question of whether a scoping review was the most suitable method versus a narrative review. The rationale for using Arksey & O’Malley’s framework should be more critically justified in light of the very small number of included studies. | While a narrative review could have provided descriptive commentary, it would not have offered the same level of methodological transparency or reproducibility as Arksey and O’Malley’s approach. By using this framework, we were able to apply a systematic and replicable search strategy, document inclusion/exclusion decisions, and present findings in a structured manner. |
| 2 | While the databases and search terms are described, it is not fully clear how inclusion/exclusion criteria were operationalized. For example: Were grey literature and unpublished reports (common in military contexts) excluded?  Were non-English studies searched beyond the specified databases? How were “military-connected children” defined (e.g., only children of active-duty members, or also veterans)? | We have added in these omitted sections into the methods section and also in the limitations of the review. This aims to add to reproducibility. |
| 2 | The use of the CASP tool is commendable. However, more detail on how the appraisal informed interpretation of findings is needed. Were any methodological weaknesses of the included studies noted (e.g., reliance on parental report, secondary data limitations)? | We have clarified CASP scores for the reviewed papers on lines 173 to 177, page 7 |
| 2 | The manuscript correctly notes the absence of children’s direct perspectives in the literature. This point could be further emphasized in the Discussion to underscore how this limits the field and to recommend methodological approaches (e.g., participatory research, child-friendly self-report tools). | We have given further detail regarding the voice of the child in lines 284-9, 253-256, 257-265. |
| 2 | The discussion highlights research gaps but could more explicitly connect findings to potential implications for military, health, and education policy. For example, how might schools or military support programs incorporate screening for ACEs/PCEs? | We have connected findings to implications for practice in the discussion section. |
| 2 | Some acronyms (e.g., CASP, PRISMA) could be spelled out on first use in the main text. | Thank you for drawing our attention to this oversight. We have amended the wording for CASP and PRISMA in the first instance. |
| 2 | Table 1 is informative but could benefit from clearer column headings and consistent formatting of examples under each category. | We have improved the clarity and description of table 1. |
| 2 | Figure 1 (PRISMA diagram) is standard but could note reasons for exclusion more succinctly. | We have updated the PRISMA diagram as suggested. |
| 2 | Several references are relatively old (e.g., Felitti, 1998). While these are foundational, more recent child resilience literature could be integrated to strengthen the BCE/PCE discussion.  The text occasionally cites reports (e.g., “Living in Our Shoes”) without sufficient contextualization for non-UK readers | We have added updated literature and expanded upon the description of the Living in our shoes report on page 4 line 95, |
| 2 | Ensure consistency in hyphenation (e.g., “military connected children” vs. “military-connected children”). | Thank you for highlighting this discrepancy in terminology. We have standardised this to military-connected children throughout the manuscript. |
