# Supplementary material for: A scoping review of Adverse, Benevolent, and Positive Childhood Experiences in military-connected children
Source: PLOS Ment Health. 2026 Jul 22;3(7):e0000654. doi: 10.1371/journal.pmen.0000654 (PMC13390850; doi:10.1371/journal.pmen.0000654)
Supplement: Attachment — Submitted filename: Comment table V2 Nov 25.docx [file pmen.0000654.s010.docx]

| Reviewer | Comment | Response |
| --- | --- | --- |
| 1 | I think that you have a well written manuscript and you have accurately reflected the lack of available literature on your selected topic. My only concern (which I will be glad to be ignored) is the overall relevancy of publishing this without a unique contribution to the identified lack. This may not fall under my purview as a reviewer, but I am struggling with accepting the manuscript because of the scope covered. I do not want this to sound like I do not value what you have written and reviewed. I just feel that it is the setup for a great piece of research. I am recommending "minor revisions" because I am completely ok with this being published if it is overall thought of as fitting the journal's mission.  The introduction provides a thorough overview of Adverse, Benevolent, and Positive Childhood Experiences (ACEs, BCEs, and PCEs), effectively situating the study within a meaningful conceptual framework. The discussion of “counter-ACEs” and protective factors is relevant and aligns with recent literature. However, the section could benefit from clearer theoretical linkage between ACEs/PCEs frameworks and the unique military context. | In response to Reviewer 1's thoughtful concern about contribution beyond gap identification, we have substantially expanded the Discussion (pages 11 and 12) to articulate why this gap has critical practical implications and to provide a research framework and methodological roadmap for addressing it. We believe this positions the review not merely as documenting absence, but as providing the conceptual and methodological foundation necessary for future empirical work in this under-researched area |
| 2 | The authors also mention use of PRISMA and CASP, which strengthens methodological transparency. However, the search strategy lacks detail: specific keywords, Boolean operators, and the rationale for excluding grey literature should be fully presented in-text rather than referring readers to appendices. | Thank you for your thoughtful comments towards publication of this manuscript. We included search strings, including Boolean operators, in table 2 and have added this to the draft manuscript.  Additionally, we have expanded on the rationale for not including grey literature pg6, line 137 - 139 |
| 2 | The screening process and data extraction procedures are briefly described but could include information on inter-rater reliability or how disagreements were resolved. | We have added a sentence to explain the screening process in further detail on page 7, lines 149-150. |
| 2 | Tables and the PRISMA flow diagram provide structure, and the narrative synthesis identifies key trends (e.g., the absence of BCE-focused studies). However, while the description of each study is useful, a synthesized summary table of findings, sample characteristics, and measures would help readers grasp cross-study patterns. | We have updated table 3 9page 9) to reflect study characteristics as suggested. Thank you for bringing this to our attention as it improves understanding of the literature. |
| 2 | The discussion provides a strong interpretive summary and appropriately identifies a paucity of research and the absence of the child’s voice in the literature. The argument for participatory, co-designed research with military-connected children and young people (MCCYP) is compelling and aligns with current child-rights perspectives (e.g., Lundy, 2007). Still, the section could benefit from a clearer link back to the aims and specific gaps identified in the results (e.g., absence of BCE studies, lack of standardized measures) | We have added a section regarding the identified gap in the literature on page 11, lines 270 to 285. This is followed by an identification of the contribution of the review to knowledge, on page 12, lines 287 to 296. Thank you for drawing this to our attention. |
