# Supplementary material for: A scoping review of Adverse, Benevolent, and Positive Childhood Experiences in military-connected children
Source: PLOS Ment Health. 2026 Jul 22;3(7):e0000654. doi: 10.1371/journal.pmen.0000654 (PMC13390850; doi:10.1371/journal.pmen.0000654)
Supplement: Attachment — Submitted filename: ACEs comment table V4.docx [file pmen.0000654.s012.docx]

| Reviewer | Comment | Response |
| --- | --- | --- |
| 2 | Overall, the revised manuscript is strong, timely, and conceptually important. It makes a clear and valuable contribution by integrating ACEs, BCEs, and PCEs within the context of military-connected children and young people, and by explicitly identifying the absence of the child’s voice in existing research. The justification for conducting a scoping review is appropriate, and the paper convincingly demonstrates that this is an emerging and underdeveloped field of inquiry. | Thank you for your considered responses to the edited manuscript and for the time you have taken. We appreciate the depth of understanding which you have on this subject and the support you have offered to guide the manuscript towards publication. |
| 2 | The title accurately reflects the scope and content of the manuscript. The abstract is generally clear and informative, but it would benefit from careful proofreading to correct typographical errors (e.g., “Mmilitary Cconnected Cchildren”). | We have edited the abstract and ensured that all tracked changes have been accepted in the clean version of the manuscript. Proofreading has been carried out across the manuscript. |
| 2 | The final sentences of the abstract are slightly repetitive in emphasising that little is known about ACEs, BCEs, and PCEs in MCCYP. These could be streamlined into a single, more concise concluding statement that highlights the unique contribution of the review, for example by explicitly stating that this is the first scoping review to map ACEs, BCEs, and PCEs research in military-connected children and young people. | We have revised the final sentences of the abstract, into concise statement which provides a stronger ending as suggested. (page 2, lines 51 and 52) |
| 2 | The conceptual framing of ACEs, BCEs, and PCEs is one of the strengths of the manuscript. The review clearly situates these constructs within the wider literature and effectively explains their relevance to resilience and developmental outcomes. However, BCEs and PCEs are sometimes treated as overlapping or interchangeable. Greater conceptual precision would strengthen the paper. It would be useful to clarify more explicitly that BCEs tend to refer to foundational experiences of safety, care, and relational stability, while PCEs often refer to experiences that actively promote growth, competence, and wellbeing. Making this distinction more explicit would strengthen the theoretical coherence of the manuscript and sharpen its contribution to the literature. | Thank you for drawing this to our attention. We have added extra sentences to this section as suggested, to give further clarity to the impact of BCEs and PCEs. (page 3, lines 74 – 74 and 78) |
| 2 | The aims of the review are clearly stated and appropriate for a scoping review. The emphasis on screening tools and self-report from children is particularly valuable. However, the methodological transparency could be enhanced by explicitly presenting two or three guiding research questions that structure the review, for example relating to the extent of existing evidence, whose perspectives dominate the literature, and what tools are used to assess ACEs, BCEs, and PCEs in military-connected populations | Thank you for your considered comment. We have not added research questions as the aim of the review was simply to find and evaluate existing research pertaining to current available literature around MCCYP experiences of ACEs, BCEs and PCEs. |
| 2 | The methods section is sound and appropriately follows Arksey and O’Malley’s framework. The database selection, inclusion and exclusion criteria, and use of CASP are well justified. The decision to exclude grey literature is reasonable given the aim of mapping peer-reviewed academic evidence, but this choice should be more explicitly acknowledged as a limitation, particularly in a field such as military and family research where high-quality reports often exist outside academic journals. A short statement recognising that important insights from policy, charities, and defence organisations may not have been captured would strengthen methodological reflexivity. | Thank you for this suggestion. We have added an extra sentence to the original text regarding grey literature in the limitations section. (page 18, lines 359- 361) |
| 2 | The results section is clear and well organised, and Table 3 is particularly helpful in synthesising study characteristics and findings. The dominance of US-based studies, reliance on parent and caregiver reports, and absence of BCE-focused research are all clearly demonstrated. When reporting CASP scores, it would strengthen the manuscript to briefly explain what high methodological quality means in practical terms, such as clarity of sampling, validity of measures, and robustness of analyses. A short paragraph explicitly summarising who is being studied, who is not being studied, and what constructs dominate the literature would further reinforce the central research gap. | Thank you for the opportunity to add to the section regarding CASP scores. We have added further detail to this section. (Page 8, lines 173 – 177) |
| 2 | The discussion section is one of the strongest parts of the manuscript. The identification of the near-total absence of child-reported data and the lack of dedicated ACE, BCE, and PCE tools for MCCYP is both convincing and important. The argument that current knowledge is filtered almost entirely through adult perspectives is clearly articulated and ethically compelling. Some repetition occurs when introducing this point, and these paragraphs could be slightly condensed to improve flow, but the core message is strong and well supported | Thank you for your kind comments regarding the discussion section. |
| 2 | The emphasis on participatory and co-produced research is particularly valuable. The discussion aligns well with children’s rights frameworks and with contemporary movements toward child-centred research design. The reference to Lundy’s model is appropriate, and the manuscript could be strengthened further by briefly linking participation not only to “voice” but also to influence, power-sharing, and co-ownership of research processes | Thank you for acknowledging the valuable nature of child centred work. We have expanded on this section to acknowledge participant voice, power and co-production. (lines 267 – 271) |
| 2 | The section on the significance and implications of the identified gap is one of the most original parts of the paper. The argument that services and policies are currently based on adult recollections or proxy reporting, rather than children’s lived experiences, is powerful and clearly articulated. This section successfully moves the paper beyond description and into policy-relevant critique. Minor tightening of language would enhance academic tone, but the content is compelling and well justified. | Thank you for your kind words regarding this section. We would like this article to be of use to people in practice in addition to academic interest and therefore have chosen to leave the wording as in previous manuscript. |
| 2 | The proposed Military Connected Childhood Experiences Framework represents an important theoretical contribution. It successfully moves the field beyond a purely deficit-based approach and recognises the coexistence of adversity and strength within military-connected childhoods. This framework would be even stronger if the authors briefly indicated how it could be used in practice, for example to guide screening tool development, inform intervention design, or shape policy frameworks. | Thank you for this important suggestion. We note that practical applications of the Military-Connected Childhood Experiences Framework, including its use to guide screening, inform intervention design, and shape policy are discussed in depth in our related work (*Watson et al., 2025, PLOS Global Public Health*), where we outline the Identify-Connect-Engage (ICE) model and its use in trauma-informed practice with military-connected children and young people. (Page 17, lines 330 -345) |
| 2 | The strengths and limitations section is well balanced and demonstrates good methodological awareness. The justification for using a scoping review is clear, and the small number of included studies is appropriately framed as a finding in itself. In addition, it would be useful to explicitly mention the geographic concentration of evidence in the United States and the limited cultural diversity of existing research as further limitations. | Thank you for highlighting this oversight. We have added a paragraph recognising these important points on page 18, lines 353 to 357. |
| 2 | The conclusion is clear, ethically grounded, and strongly aligned with participatory research principles. The call to move from research conducted “on” MCCYP to research conducted “with” MCCYP is particularly effective. The conclusion could be slightly tightened by reducing repetition and ending with a concise, forward-looking statement that emphasises the need for balanced screening approaches addressing both adversity and strength. | Thank you for this comment. We have amended the conclusion and added a brief statement at the end of the section to highlight future focus and summarise the manuscripts outcomes. |
| 2 | Finally, the manuscript would benefit from careful proofreading to correct typographical and formatting errors, including repeated words, inconsistent capitalisation of ACEs, BCEs, and PCEs, and occasional awkward phrasing. Addressing these minor issues will improve the overall polish of the manuscript and strengthen its readiness for publication | Thank you for your guidance with this manuscript. We have proofread the manuscript to address the minor issues which you highlighted. |
