# Supplementary material for: A scoping review of Adverse, Benevolent, and Positive Childhood Experiences in military-connected children
Source: PLOS Ment Health. 2026 Jul 22;3(7):e0000654. doi: 10.1371/journal.pmen.0000654 (PMC13390850; doi:10.1371/journal.pmen.0000654)
Supplement: Attachment — Submitted filename: Comment table ACEs V5 .docx [file pmen.0000654.s013.docx]

| Reviewer | Comment | Response |
| --- | --- | --- |
| 2 | The authors can consider refining the review question to focus more explicitly on mapping evidence on ACEs, BCEs, and PCEs among MCCYP, with child self-report treated as a secondary analytical observation rather than a core aim. | We are grateful for your work on supporting the publication of this manuscript. As per scoping review guidance, this question was formulated prior to any searches being carried out. Any amendment to the question would change the underpinning aims of completing this review, and the original scope of the project. |
| 2 | Relevant studies may have been excluded simply because they did not explicitly use ACE/BCE/PCE terminology, coming from different terminology used in other studies. The authors can consider acknowledging this limitation more explicitly and discussing how conceptual synonyms or related constructs may exist in the literature but were not captured by the search strategy. | We have added a sentence on page 18/19, lines 373 – 376 to indicate which literature may have been excluded due to not specifically using the terminology specified by this review (ACEs, BCEs or PCEs). |
| 2 | The authors can consider discussing more explicitly how excluding grey literature may have underestimated the available evidence base, particularly in military family research where policy reports are common. | Page 18, lines 367 to 374 include a description of the rationale and impact of exclusion of grey literature from this review. |
| 2 | The authors can consider clarifying: whether independent dual screening occurred whether inter-reviewer agreement was assessed how discrepancies were resolved. | Thank you for highlighting this omission. We have added more wording to indicate the review process on page 7, lines 150 -151. |
| 2 | The authors can consider clarifying: whether CASP was used for descriptive appraisal rather than scoring, and how study quality influenced interpretation of the findings. | We have included more information about this process on page 8, lines 154 – 158. |
| 2 | The authors can consider expanding the conceptual explanation of how BCEs and PCEs differ theoretically and empirically, and why both constructs are necessary in this review. | Thank you for bringing this to our attention. We have clarified our inclusion of both concepts on page ¾, lines 81 – 85. |
| 2 | The authors can consider further tempering conclusions, emphasising that findings primarily reflect US-based military systems and datasets. | Thank you for this suggestion. We refer to the over inclusion of US literature in the results section, and have added a sentence to the conclusion to link to this (page 19, lines 390 – 392) |
| 2 | The manuscript still includes “table ##” in the methods section. Other typographical errors also exist. So proofreading is needed. | We have amended the use of ## in the methods section and have carefully proofread the manuscript. |
| 2 | The authors can consider softening the claim to “one of the first reviews…” or “the first review explicitly examining ACEs, BCEs, and PCEs together…”. | Thank you for this suggestion, we have utilised the second suggested sentence to soften the claim (lines 52/53, page 2). |
