# Supplementary material for: A scoping review of Adverse, Benevolent, and Positive Childhood Experiences in military-connected children
Source: PLOS Ment Health. 2026 Jul 22;3(7):e0000654. doi: 10.1371/journal.pmen.0000654 (PMC13390850; doi:10.1371/journal.pmen.0000654)
Supplement: Peer review history

# Peer review: Decision Letter 0

**DOI:** 10.1371/journal.pmen.0000654.r001

**Authors:** Gellan Karamallah Ramadan Ahmed

4 Sep 2025

PMEN-D-25-00297

A Scoping Review of Adverse, Benevolent, and Positive Childhood Experiences in Military-Connected Children

PLOS Mental Health

Dear Dr. Mason,

Thank you for submitting your manuscript to PLOS Mental Health. After careful consideration, we feel that it has merit but does not fully meet PLOS Mental Health’s publication criteria as it currently stands. Therefore, we invite you to submit a revised version of the manuscript that addresses the points raised during the review process.

Please submit your revised manuscript by Oct 19 2025 11:59PM. If you will need more time than this to complete your revisions, please reply to this message or contact the journal office at mentalhealth@plos.org. When you're ready to submit your revision, log on to https://www.editorialmanager.com/pmen/ and select the 'Submissions Needing Revision' folder to locate your manuscript file.

Please include the following items when submitting your revised manuscript:

- A rebuttal letter that responds to each point raised by the editor and reviewer(s). You should upload this letter as a separate file labeled 'Response to Reviewers'.
- A marked-up copy of your manuscript that highlights changes made to the original version. You should upload this as a separate file labeled 'Revised Manuscript with Track Changes'.
- An unmarked version of your revised paper without tracked changes. You should upload this as a separate file labeled 'Manuscript'.

Guidelines for resubmitting your figure files are available below the reviewer comments at the end of this letter.

We look forward to receiving your revised manuscript.

Kind regards,

Gellan Karamallah Ramadan Ahmed

Academic Editor

PLOS Mental Health

Journal Requirements:

If the reviewer comments include a recommendation to cite specific previously published works, please review and evaluate these publications to determine whether they are relevant and should be cited. There is no requirement to cite these works unless the editor has indicated otherwise.

Additional Editor Comments (if provided):

Reviewer #1:

Reviewer #2:

[Note: HTML markup is below. Please do not edit.]

Reviewers' comments:

Reviewer's Responses to Questions

**Comments to the Author**

1. Does this manuscript meet PLOS Mental Health’s publication criteria? Is the manuscript technically sound, and do the data support the conclusions? The manuscript must describe methodologically and ethically rigorous research with conclusions that are appropriately drawn based on the data presented.

Reviewer #1: Yes

Reviewer #2: Yes

**********

2. Has the statistical analysis been performed appropriately and rigorously?

Reviewer #1: Yes

Reviewer #2: N/A

**********

3. Have the authors made all data underlying the findings in their manuscript fully available (please refer to the Data Availability Statement at the start of the manuscript PDF file)?

The PLOS Data policy requires authors to make all data underlying the findings described in their manuscript fully available without restriction, with rare exception. The data should be provided as part of the manuscript or its supporting information, or deposited to a public repository. For example, in addition to summary statistics, the data points behind means, medians and variance measures should be available. If there are restrictions on publicly sharing data—e.g. participant privacy or use of data from a third party—those must be specified.

Reviewer #1: Yes

Reviewer #2: Yes

**********

4. Is the manuscript presented in an intelligible fashion and written in standard English?

PLOS Mental Health does not copyedit accepted manuscripts, so the language in submitted articles must be clear, correct, and unambiguous. Any typographical or grammatical errors should be corrected at revision, so please note any specific errors here.

Reviewer #1: Yes

Reviewer #2: Yes

**********

5. Review Comments to the Author

Please use the space provided to explain your answers to the questions above. You may also include additional comments for the author, including concerns about dual publication, research ethics, or publication ethics. (Please upload your review as an attachment if it exceeds 20,000 characters)

Reviewer #1: While I appreciate the concepts presented in the manuscript, the conclusions do not extend past the idea that there is a lack of literature regarding the topic. While I have no doubt that this is the case, it is not just additional research that is required, but rather research period is required to understand this.

Reviewer #2: Conceptual Clarity (ACEs, BCEs, PCEs):

The manuscript provides useful definitions of ACEs, BCEs, and PCEs, but at times these categories are blurred. For instance, the distinction between BCEs and PCEs is not consistently sustained throughout the paper. Clarifying how these differ and why both are included (rather than subsumed as “counter-ACEs”) would enhance theoretical coherence.

Scope and Aims of the Review:

The stated aim is to “map existing literature and gain a deeper understanding of the current landscape of ACEs, BCEs, and PCEs in military-connected children.” However, only six studies were identified, and none directly addressed BCEs. This raises the question of whether a scoping review was the most suitable method versus a narrative review. The rationale for using Arksey & O’Malley’s framework should be more critically justified in light of the very small number of included studies.

Methodological Transparency:

While the databases and search terms are described, it is not fully clear how inclusion/exclusion criteria were operationalized. For example:

Were grey literature and unpublished reports (common in military contexts) excluded?

Were non-English studies searched beyond the specified databases?

How were “military-connected children” defined (e.g., only children of active-duty members, or also veterans)?

Clarification would enhance reproducibility.

Quality Appraisal:

The use of the CASP tool is commendable. However, more detail on how the appraisal informed interpretation of findings is needed. Were any methodological weaknesses of the included studies noted (e.g., reliance on parental report, secondary data limitations)?

Voice of the Child:

The manuscript correctly notes the absence of children’s direct perspectives in the literature. This point could be further emphasized in the Discussion to underscore how this limits the field and to recommend methodological approaches (e.g., participatory research, child-friendly self-report tools).

Contribution to Policy and Practice:

The discussion highlights research gaps but could more explicitly connect findings to potential implications for military, health, and education policy. For example, how might schools or military support programs incorporate screening for ACEs/PCEs?

Terminology:

Throughout, “military connected children” and “military-connected children” are used inconsistently. Standardize to “military-connected.”

Some acronyms (e.g., CASP, PRISMA) could be spelled out on first use in the main text.

Presentation of Tables and Figures:

Table 1 is informative but could benefit from clearer column headings and consistent formatting of examples under each category.

Figure 1 (PRISMA diagram) is standard but could note reasons for exclusion more succinctly.

Citations:

Several references are relatively old (e.g., Felitti, 1998). While these are foundational, more recent child resilience literature could be integrated to strengthen the BCE/PCE discussion.

The text occasionally cites reports (e.g., “Living in Our Shoes”) without sufficient contextualization for non-UK readers.

The manuscript is generally well-written, but some sentences could be tightened for clarity. For example:

“Military-connected children and young people face unique stressors…” could be streamlined to “Military-connected children face unique stressors…”

Ensure consistency in hyphenation (e.g., “military connected children” vs. “military-connected children”).

**********

6. PLOS authors have the option to publish the peer review history of their article (what does this mean?). If published, this will include your full peer review and any attached files.

**Do you want your identity to be public for this peer review?**  If you choose “no”, your identity will remain anonymous but your review may still be made public.

For information about this choice, including consent withdrawal, please see our Privacy Policy.

Reviewer #1: No

Reviewer #2: **Yes:** Peejay D. Bengwasan

**********

[NOTE: If reviewer comments were submitted as an attachment file, they will be attached to this email and accessible via the submission site. Please log into your account, locate the manuscript record, and check for the action link "View Attachments". If this link does not appear, there are no attachment files.]

While revising your submission, please upload your figure files to the Preflight Analysis and Conversion Engine (PACE) digital diagnostic tool, https://pacev2.apexcovantage.com/. PACE helps ensure that figures meet PLOS requirements. To use PACE, you must first register as a user. Registration is free. Then, login and navigate to the UPLOAD tab, where you will find detailed instructions on how to use the tool. If you encounter any issues or have any questions when using PACE, please email PLOS at figures@plos.org. Please note that Supporting Information files do not need this step.

# Author comment: Author response to Decision Letter 1

**DOI:** 10.1371/journal.pmen.0000654.r002

13 Oct 2025

Attachment Submitted filename: ACEs comment table October 25.docx

# Peer review: Decision Letter 1

**DOI:** 10.1371/journal.pmen.0000654.r003

**Authors:** Gellan Karamallah Ramadan Ahmed

4 Nov 2025

PMEN-D-25-00297R1

A Scoping Review of Adverse, Benevolent, and Positive Childhood Experiences in Military-Connected Children

PLOS Mental Health

Dear Dr. Mason,

Thank you for submitting your manuscript to PLOS Mental Health. After careful consideration, we feel that it has merit but does not fully meet PLOS Mental Health’s publication criteria as it currently stands. Therefore, we invite you to submit a revised version of the manuscript that addresses the points raised during the review process.

Please submit your revised manuscript by Dec 04 2025 11:59PM. If you will need more time than this to complete your revisions, please reply to this message or contact the journal office at mentalhealth@plos.org. When you're ready to submit your revision, log on to https://www.editorialmanager.com/pmen/ and select the 'Submissions Needing Revision' folder to locate your manuscript file.

Please include the following items when submitting your revised manuscript:

- A rebuttal letter that responds to each point raised by the editor and reviewer(s). You should upload this letter as a separate file labeled 'Response to Reviewers'.
- A marked-up copy of your manuscript that highlights changes made to the original version. You should upload this as a separate file labeled 'Revised Manuscript with Track Changes'.
- An unmarked version of your revised paper without tracked changes. You should upload this as a separate file labeled 'Manuscript'.

Guidelines for resubmitting your figure files are available below the reviewer comments at the end of this letter.

We look forward to receiving your revised manuscript.

Kind regards,

Gellan Karamallah Ramadan Ahmed

Academic Editor

PLOS Mental Health

Journal Requirements:

If the reviewer comments include a recommendation to cite specific previously published works, please review and evaluate these publications to determine whether they are relevant and should be cited. There is no requirement to cite these works unless the editor has indicated otherwise.

Please review your reference list to ensure that it is complete and correct. If you have cited papers that have been retracted, please include the rationale for doing so in the manuscript text, or remove these references and replace them with relevant current references. Any changes to the reference list should be mentioned in the rebuttal letter that accompanies your revised manuscript. If you need to cite a retracted article, indicate the article’s retracted status in the References list and also include a citation and full reference for the retraction notice.

Additional Editor Comments (if provided):

[Note: HTML markup is below. Please do not edit.]

Reviewers' comments:

Reviewer's Responses to Questions

**Comments to the Author**

1. If the authors have adequately addressed your comments raised in a previous round of review and you feel that this manuscript is now acceptable for publication, you may indicate that here to bypass the “Comments to the Author” section, enter your conflict of interest statement in the “Confidential to Editor” section, and submit your "Accept" recommendation.

Reviewer #1: All comments have been addressed

Reviewer #2: All comments have been addressed

**********

2. Does this manuscript meet PLOS Mental Health’s publication criteria? Is the manuscript technically sound, and do the data support the conclusions? The manuscript must describe methodologically and ethically rigorous research with conclusions that are appropriately drawn based on the data presented.

Reviewer #1: Partly

Reviewer #2: (No Response)

**********

3. Has the statistical analysis been performed appropriately and rigorously?

Reviewer #1: N/A

Reviewer #2: Yes

**********

4. Have the authors made all data underlying the findings in their manuscript fully available (please refer to the Data Availability Statement at the start of the manuscript PDF file)?

The PLOS Data policy requires authors to make all data underlying the findings described in their manuscript fully available without restriction, with rare exception. The data should be provided as part of the manuscript or its supporting information, or deposited to a public repository. For example, in addition to summary statistics, the data points behind means, medians and variance measures should be available. If there are restrictions on publicly sharing data—e.g. participant privacy or use of data from a third party—those must be specified.

Reviewer #1: Yes

Reviewer #2: Yes

**********

5. Is the manuscript presented in an intelligible fashion and written in standard English?

PLOS Mental Health does not copyedit accepted manuscripts, so the language in submitted articles must be clear, correct, and unambiguous. Any typographical or grammatical errors should be corrected at revision, so please note any specific errors here.

Reviewer #1: Yes

Reviewer #2: Yes

**********

6. Review Comments to the Author

Please use the space provided to explain your answers to the questions above. You may also include additional comments for the author, including concerns about dual publication, research ethics, or publication ethics. (Please upload your review as an attachment if it exceeds 20,000 characters)

Reviewer #1: I think that you have a well written manuscript and you have accurately reflected the lack of available literature on your selected topic. My only concern (which I will be glad to be ignored) is the overall relevancy of publishing this without a unique contribution to the identified lack. This may not fall under my purview as a reviewer, but I am struggling with accepting the manuscript because of the scope covered. I do not want this to sound like I do not value what you have written and reviewed. I just feel that it is the setup for a great piece of research. I am recommending "minor revisions" because I am completely ok with this being published if it is overall thought of as fitting the journal's mission.

Reviewer #2: The introduction provides a thorough overview of Adverse, Benevolent, and Positive Childhood Experiences (ACEs, BCEs, and PCEs), effectively situating the study within a meaningful conceptual framework. The discussion of “counter-ACEs” and protective factors is relevant and aligns with recent literature. However, the section could benefit from clearer theoretical linkage between ACEs/PCEs frameworks and the unique military context.

The authors also mention use of PRISMA and CASP, which strengthens methodological transparency. However, the search strategy lacks detail: specific keywords, Boolean operators, and the rationale for excluding grey literature should be fully presented in-text rather than referring readers to appendices.

The screening process and data extraction procedures are briefly described but could include information on inter-rater reliability or how disagreements were resolved.

Tables and the PRISMA flow diagram provide structure, and the narrative synthesis identifies key trends (e.g., the absence of BCE-focused studies). However, while the description of each study is useful, a synthesized summary table of findings, sample characteristics, and measures would help readers grasp cross-study patterns.

The discussion provides a strong interpretive summary and appropriately identifies a paucity of research and the absence of the child’s voice in the literature. The argument for participatory, co-designed research with military-connected children and young people (MCCYP) is compelling and aligns with current child-rights perspectives (e.g., Lundy, 2007). Still, the section could benefit from a clearer link back to the aims and specific gaps identified in the results (e.g., absence of BCE studies, lack of standardized measures).

**********

7. PLOS authors have the option to publish the peer review history of their article (what does this mean?). If published, this will include your full peer review and any attached files.

**Do you want your identity to be public for this peer review?**  If you choose “no”, your identity will remain anonymous but your review may still be made public.

For information about this choice, including consent withdrawal, please see our Privacy Policy.

Reviewer #1: No

Reviewer #2: **Yes:** Peejay D. Bengwasan

**********

[NOTE: If reviewer comments were submitted as an attachment file, they will be attached to this email and accessible via the submission site. Please log into your account, locate the manuscript record, and check for the action link "View Attachments". If this link does not appear, there are no attachment files.]

-->Figure Resubmissions:

While revising your submission, we strongly recommend that you use PLOS’s NAAS tool (https://ngplosjournals.pagemajik.ai/artanalysis) to test your figure files. NAAS can convert your figure files to the TIFF file type and meet basic requirements (such as print size, resolution), or provide you with a report on issues that do not meet our requirements and that NAAS cannot fix.

After uploading your figures to PLOS’s NAAS tool - https://ngplosjournals.pagemajik.ai/artanalysis, NAAS will process the files provided and display the results in the "Uploaded Files" section of the page as the processing is complete. If the uploaded figures meet our requirements (or NAAS is able to fix the files to meet our requirements), the figure will be marked as "fixed" above. If NAAS is unable to fix the files, a red "failed" label will appear above. When NAAS has confirmed that the figure files meet our requirements, please download the file via the download option, and include these NAAS processed figure files when submitting your revised manuscript.

# Author comment: Author response to Decision Letter 2

**DOI:** 10.1371/journal.pmen.0000654.r004

2 Dec 2025

Attachment Submitted filename: Comment table V2 Nov 25.docx

# Peer review: Decision Letter 2

**DOI:** 10.1371/journal.pmen.0000654.r005

**Authors:** Gellan Karamallah Ramadan Ahmed

16 Dec 2025

PMEN-D-25-00297R2

A Scoping Review of Adverse, Benevolent, and Positive Childhood Experiences in Military-Connected Children

PLOS Mental Health

Dear Dr. Mason,

Thank you for submitting your manuscript to PLOS Mental Health. After careful consideration, we feel that it has merit but does not fully meet PLOS Mental Health’s publication criteria as it currently stands. Therefore, we invite you to submit a revised version of the manuscript that addresses the points raised during the review process.

Please submit your revised manuscript by Jan 15 2026 11:59PM. If you will need more time than this to complete your revisions, please reply to this message or contact the journal office at mentalhealth@plos.org. When you're ready to submit your revision, log on to https://www.editorialmanager.com/pmen/ and select the 'Submissions Needing Revision' folder to locate your manuscript file.

Please include the following items when submitting your revised manuscript:

- A letter that responds to each point raised by the editor and reviewer(s). You should upload this letter as a separate file labeled 'Response to Reviewers'.
- A marked-up copy of your manuscript that highlights changes made to the original version. You should upload this as a separate file labeled 'Revised Manuscript with Track Changes'.
- An unmarked version of your revised paper without tracked changes. You should upload this as a separate file labeled 'Manuscript'.

Guidelines for resubmitting your figure files are available below the reviewer comments at the end of this letter.

We look forward to receiving your revised manuscript.

Kind regards,

Gellan Karamallah Ramadan Ahmed

Academic Editor

PLOS Mental Health

Journal Requirements:

If the reviewer comments include a recommendation to cite specific previously published works, please review and evaluate these publications to determine whether they are relevant and should be cited. There is no requirement to cite these works unless the editor has indicated otherwise.

Please review your reference list to ensure that it is complete and correct. If you have cited papers that have been retracted, please include the rationale for doing so in the manuscript text, or remove these references and replace them with relevant current references. Any changes to the reference list should be mentioned in the rebuttal letter that accompanies your revised manuscript. If you need to cite a retracted article, indicate the article’s retracted status in the References list and also include a citation and full reference for the retraction notice.

Additional Editor Comments (if provided):

[Note: HTML markup is below. Please do not edit.]

Reviewers' comments:

Reviewer's Responses to Questions

**Comments to the Author**

1. If the authors have adequately addressed your comments raised in a previous round of review and you feel that this manuscript is now acceptable for publication, you may indicate that here to bypass the “Comments to the Author” section, enter your conflict of interest statement in the “Confidential to Editor” section, and submit your "Accept" recommendation.

Reviewer #1: All comments have been addressed

Reviewer #2: All comments have been addressed

**********

2. Does this manuscript meet PLOS Mental Health’s publication criteria? Is the manuscript technically sound, and do the data support the conclusions? The manuscript must describe methodologically and ethically rigorous research with conclusions that are appropriately drawn based on the data presented.

Reviewer #1: Partly

Reviewer #2: Yes

**********

3. Has the statistical analysis been performed appropriately and rigorously?

Reviewer #1: N/A

Reviewer #2: Yes

**********

4. Have the authors made all data underlying the findings in their manuscript fully available (please refer to the Data Availability Statement at the start of the manuscript PDF file)?

The PLOS Data policy requires authors to make all data underlying the findings described in their manuscript fully available without restriction, with rare exception. The data should be provided as part of the manuscript or its supporting information, or deposited to a public repository. For example, in addition to summary statistics, the data points behind means, medians and variance measures should be available. If there are restrictions on publicly sharing data—e.g. participant privacy or use of data from a third party—those must be specified.

Reviewer #1: Yes

Reviewer #2: Yes

**********

5. Is the manuscript presented in an intelligible fashion and written in standard English?

PLOS Mental Health does not copyedit accepted manuscripts, so the language in submitted articles must be clear, correct, and unambiguous. Any typographical or grammatical errors should be corrected at revision, so please note any specific errors here.

Reviewer #1: Yes

Reviewer #2: Yes

**********

6. Review Comments to the Author

Please use the space provided to explain your answers to the questions above. You may also include additional comments for the author, including concerns about dual publication, research ethics, or publication ethics. (Please upload your review as an attachment if it exceeds 20,000 characters)

Reviewer #1: It seems as if the authors have addressed the concerns of other reviewers. I, again, stand by my original assertion that if this meets the criteria for publishing from the journal I am in support of publishing it. My original concern had nothing to do with the writing/analysis of the authors, but rather a concern or question about the overall application of the content. I am in support of the editor's decision.

Reviewer #2: Given the very small number of included studies (n = 6), the Results section occasionally reads more like an extended summary of individual papers. Consider synthesising findings more explicitly around themes (e.g., measurement gaps, informant bias, intergenerational trauma).

The Results section could more clearly separate descriptive findings (e.g., study characteristics) from substantive findings (e.g., patterns of ACE exposure, buffering effects of PCEs).

The discussion section is relatively long and could benefit from tighter structuring with clearer subheadings or more concise synthesis in some sections.

Some arguments (e.g., the urgency of participatory research, limitations of adult-reported data) are repeated across multiple subsections and could be streamlined.

While strengths and limitations are addressed thoroughly, the implications for clinical practice, education systems, and military family services could be more explicitly articulated.

This manuscript addresses an important and under-researched area and makes a meaningful contribution by mapping the near-absence of BCEs and PCEs research and especially child-reported data among military-connected children. With some tightening of the introduction and discussion, clearer articulation of research questions, and stronger synthesis in the Results section, the paper would be well positioned for publication in a mental health journal with an applied and policy-oriented readership.

**********

7. PLOS authors have the option to publish the peer review history of their article (what does this mean?). If published, this will include your full peer review and any attached files.

**Do you want your identity to be public for this peer review?**  If you choose “no”, your identity will remain anonymous but your review may still be made public.

For information about this choice, including consent withdrawal, please see our Privacy Policy.

Reviewer #1: No

Reviewer #2: **Yes:** Peejay D. Bengwasan

**********

[NOTE: If reviewer comments were submitted as an attachment file, they will be attached to this email and accessible via the submission site. Please log into your account, locate the manuscript record, and check for the action link "View Attachments". If this link does not appear, there are no attachment files.]

-->Figure Resubmissions:

While revising your submission, we strongly recommend that you use PLOS’s NAAS tool (https://ngplosjournals.pagemajik.ai/artanalysis) to test your figure files. NAAS can convert your figure files to the TIFF file type and meet basic requirements (such as print size, resolution), or provide you with a report on issues that do not meet our requirements and that NAAS cannot fix.

After uploading your figures to PLOS’s NAAS tool - https://ngplosjournals.pagemajik.ai/artanalysis, NAAS will process the files provided and display the results in the "Uploaded Files" section of the page as the processing is complete. If the uploaded figures meet our requirements (or NAAS is able to fix the files to meet our requirements), the figure will be marked as "fixed" above. If NAAS is unable to fix the files, a red "failed" label will appear above. When NAAS has confirmed that the figure files meet our requirements, please download the file via the download option, and include these NAAS processed figure files when submitting your revised manuscript.

# Author comment: Author response to Decision Letter 3

**DOI:** 10.1371/journal.pmen.0000654.r006

13 Jan 2026

Attachment Submitted filename: Comment table V3 Jan 26.docx

# Peer review: Decision Letter 3

**DOI:** 10.1371/journal.pmen.0000654.r007

**Authors:** Gellan Karamallah Ramadan Ahmed

29 Jan 2026

PMEN-D-25-00297R3

A Scoping Review of Adverse, Benevolent, and Positive Childhood Experiences in Military-Connected Children

PLOS Mental Health

Dear Dr. Mason,

Thank you for submitting your manuscript to PLOS Mental Health. After careful consideration, we feel that it has merit but does not fully meet PLOS Mental Health’s publication criteria as it currently stands. Therefore, we invite you to submit a revised version of the manuscript that addresses the points raised during the review process.

Please submit your revised manuscript by Feb 28 2026 11:59PM. If you will need more time than this to complete your revisions, please reply to this message or contact the journal office at mentalhealth@plos.org. When you're ready to submit your revision, log on to https://www.editorialmanager.com/pmen/ and select the 'Submissions Needing Revision' folder to locate your manuscript file.

Please include the following items when submitting your revised manuscript:

- A letter that responds to each point raised by the editor and reviewer(s). You should upload this letter as a separate file labeled 'Response to Reviewers'.
- A marked-up copy of your manuscript that highlights changes made to the original version. You should upload this as a separate file labeled 'Revised Manuscript with Track Changes'.
- An unmarked version of your revised paper without tracked changes. You should upload this as a separate file labeled 'Manuscript'.

Guidelines for resubmitting your figure files are available below the reviewer comments at the end of this letter.

We look forward to receiving your revised manuscript.

Kind regards,

Gellan Karamallah Ramadan Ahmed

Academic Editor

PLOS Mental Health

Journal Requirements:

If the reviewer comments include a recommendation to cite specific previously published works, please review and evaluate these publications to determine whether they are relevant and should be cited. There is no requirement to cite these works unless the editor has indicated otherwise.

Please review your reference list to ensure that it is complete and correct. If you have cited papers that have been retracted, please include the rationale for doing so in the manuscript text, or remove these references and replace them with relevant current references. Any changes to the reference list should be mentioned in the rebuttal letter that accompanies your revised manuscript. If you need to cite a retracted article, indicate the article’s retracted status in the References list and also include a citation and full reference for the retraction notice.

Additional Editor Comments (if provided):

[Note: HTML markup is below. Please do not edit.]

Reviewers' comments:

Reviewer's Responses to Questions

**Comments to the Author**

1. If the authors have adequately addressed your comments raised in a previous round of review and you feel that this manuscript is now acceptable for publication, you may indicate that here to bypass the “Comments to the Author” section, enter your conflict of interest statement in the “Confidential to Editor” section, and submit your "Accept" recommendation.

Reviewer #2: (No Response)

**********

2. Does this manuscript meet PLOS Mental Health’s publication criteria? Is the manuscript technically sound, and do the data support the conclusions? The manuscript must describe methodologically and ethically rigorous research with conclusions that are appropriately drawn based on the data presented.

Reviewer #2: (No Response)

**********

3. Has the statistical analysis been performed appropriately and rigorously?

Reviewer #2: (No Response)

**********

4. Have the authors made all data underlying the findings in their manuscript fully available (please refer to the Data Availability Statement at the start of the manuscript PDF file)?

The PLOS Data policy requires authors to make all data underlying the findings described in their manuscript fully available without restriction, with rare exception. The data should be provided as part of the manuscript or its supporting information, or deposited to a public repository. For example, in addition to summary statistics, the data points behind means, medians and variance measures should be available. If there are restrictions on publicly sharing data—e.g. participant privacy or use of data from a third party—those must be specified.

Reviewer #2: (No Response)

**********

5. Is the manuscript presented in an intelligible fashion and written in standard English?

PLOS Mental Health does not copyedit accepted manuscripts, so the language in submitted articles must be clear, correct, and unambiguous. Any typographical or grammatical errors should be corrected at revision, so please note any specific errors here.

Reviewer #2: (No Response)

**********

6. Review Comments to the Author

Please use the space provided to explain your answers to the questions above. You may also include additional comments for the author, including concerns about dual publication, research ethics, or publication ethics. (Please upload your review as an attachment if it exceeds 20,000 characters)

Reviewer #2: Overall, the revised manuscript is strong, timely, and conceptually important. It makes a clear and valuable contribution by integrating ACEs, BCEs, and PCEs within the context of military-connected children and young people, and by explicitly identifying the absence of the child’s voice in existing research. The justification for conducting a scoping review is appropriate, and the paper convincingly demonstrates that this is an emerging and underdeveloped field of inquiry.

The title accurately reflects the scope and content of the manuscript. The abstract is generally clear and informative, but it would benefit from careful proofreading to correct typographical errors (e.g., “Mmilitary Cconnected Cchildren”). The final sentences of the abstract are slightly repetitive in emphasising that little is known about ACEs, BCEs, and PCEs in MCCYP. These could be streamlined into a single, more concise concluding statement that highlights the unique contribution of the review, for example by explicitly stating that this is the first scoping review to map ACEs, BCEs, and PCEs research in military-connected children and young people.

The conceptual framing of ACEs, BCEs, and PCEs is one of the strengths of the manuscript. The review clearly situates these constructs within the wider literature and effectively explains their relevance to resilience and developmental outcomes. However, BCEs and PCEs are sometimes treated as overlapping or interchangeable. Greater conceptual precision would strengthen the paper. It would be useful to clarify more explicitly that BCEs tend to refer to foundational experiences of safety, care, and relational stability, while PCEs often refer to experiences that actively promote growth, competence, and wellbeing. Making this distinction more explicit would strengthen the theoretical coherence of the manuscript and sharpen its contribution to the literature.

The aims of the review are clearly stated and appropriate for a scoping review. The emphasis on screening tools and self-report from children is particularly valuable. However, the methodological transparency could be enhanced by explicitly presenting two or three guiding research questions that structure the review, for example relating to the extent of existing evidence, whose perspectives dominate the literature, and what tools are used to assess ACEs, BCEs, and PCEs in military-connected populations.

The methods section is sound and appropriately follows Arksey and O’Malley’s framework. The database selection, inclusion and exclusion criteria, and use of CASP are well justified. The decision to exclude grey literature is reasonable given the aim of mapping peer-reviewed academic evidence, but this choice should be more explicitly acknowledged as a limitation, particularly in a field such as military and family research where high-quality reports often exist outside academic journals. A short statement recognising that important insights from policy, charities, and defence organisations may not have been captured would strengthen methodological reflexivity.

The results section is clear and well organised, and Table 3 is particularly helpful in synthesising study characteristics and findings. The dominance of US-based studies, reliance on parent and caregiver reports, and absence of BCE-focused research are all clearly demonstrated. When reporting CASP scores, it would strengthen the manuscript to briefly explain what high methodological quality means in practical terms, such as clarity of sampling, validity of measures, and robustness of analyses. A short paragraph explicitly summarising who is being studied, who is not being studied, and what constructs dominate the literature would further reinforce the central research gap.

The discussion section is one of the strongest parts of the manuscript. The identification of the near-total absence of child-reported data and the lack of dedicated ACE, BCE, and PCE tools for MCCYP is both convincing and important. The argument that current knowledge is filtered almost entirely through adult perspectives is clearly articulated and ethically compelling. Some repetition occurs when introducing this point, and these paragraphs could be slightly condensed to improve flow, but the core message is strong and well supported.

The emphasis on participatory and co-produced research is particularly valuable. The discussion aligns well with children’s rights frameworks and with contemporary movements toward child-centred research design. The reference to Lundy’s model is appropriate, and the manuscript could be strengthened further by briefly linking participation not only to “voice” but also to influence, power-sharing, and co-ownership of research processes.

The section on the significance and implications of the identified gap is one of the most original parts of the paper. The argument that services and policies are currently based on adult recollections or proxy reporting, rather than children’s lived experiences, is powerful and clearly articulated. This section successfully moves the paper beyond description and into policy-relevant critique. Minor tightening of language would enhance academic tone, but the content is compelling and well justified.

The proposed Military Connected Childhood Experiences Framework represents an important theoretical contribution. It successfully moves the field beyond a purely deficit-based approach and recognises the coexistence of adversity and strength within military-connected childhoods. This framework would be even stronger if the authors briefly indicated how it could be used in practice, for example to guide screening tool development, inform intervention design, or shape policy frameworks.

The strengths and limitations section is well balanced and demonstrates good methodological awareness. The justification for using a scoping review is clear, and the small number of included studies is appropriately framed as a finding in itself. In addition, it would be useful to explicitly mention the geographic concentration of evidence in the United States and the limited cultural diversity of existing research as further limitations.

The conclusion is clear, ethically grounded, and strongly aligned with participatory research principles. The call to move from research conducted “on” MCCYP to research conducted “with” MCCYP is particularly effective. The conclusion could be slightly tightened by reducing repetition and ending with a concise, forward-looking statement that emphasises the need for balanced screening approaches addressing both adversity and strength.

Finally, the manuscript would benefit from careful proofreading to correct typographical and formatting errors, including repeated words, inconsistent capitalisation of ACEs, BCEs, and PCEs, and occasional awkward phrasing. Addressing these minor issues will improve the overall polish of the manuscript and strengthen its readiness for publication.

**********

7. PLOS authors have the option to publish the peer review history of their article (what does this mean?). If published, this will include your full peer review and any attached files.

**Do you want your identity to be public for this peer review?**  If you choose “no”, your identity will remain anonymous but your review may still be made public.

For information about this choice, including consent withdrawal, please see our Privacy Policy.

Reviewer #2: **Yes:** Peejay D. Bengwasan

**********

[NOTE: If reviewer comments were submitted as an attachment file, they will be attached to this email and accessible via the submission site. Please log into your account, locate the manuscript record, and check for the action link "View Attachments". If this link does not appear, there are no attachment files.]

-->Figure Resubmissions:

While revising your submission, we strongly recommend that you use PLOS’s NAAS tool (https://ngplosjournals.pagemajik.ai/artanalysis) to test your figure files. NAAS can convert your figure files to the TIFF file type and meet basic requirements (such as print size, resolution), or provide you with a report on issues that do not meet our requirements and that NAAS cannot fix.

After uploading your figures to PLOS’s NAAS tool - https://ngplosjournals.pagemajik.ai/artanalysis, NAAS will process the files provided and display the results in the "Uploaded Files" section of the page as the processing is complete. If the uploaded figures meet our requirements (or NAAS is able to fix the files to meet our requirements), the figure will be marked as "fixed" above. If NAAS is unable to fix the files, a red "failed" label will appear above. When NAAS has confirmed that the figure files meet our requirements, please download the file via the download option, and include these NAAS processed figure files when submitting your revised manuscript.

# Author comment: Author response to Decision Letter 4

**DOI:** 10.1371/journal.pmen.0000654.r008

23 Feb 2026

Attachment Submitted filename: ACEs comment table V4.docx

# Peer review: Decision Letter 4

**DOI:** 10.1371/journal.pmen.0000654.r009

**Authors:** Gellan Karamallah Ramadan Ahmed

22 Mar 2026

PMEN-D-25-00297R4

A Scoping Review of Adverse, Benevolent, and Positive Childhood Experiences in Military-Connected Children

PLOS Mental Health

Dear Dr. Mason,

Thank you for submitting your manuscript to PLOS Mental Health. After careful consideration, we feel that it has merit but does not fully meet PLOS Mental Health’s publication criteria as it currently stands. Therefore, we invite you to submit a revised version of the manuscript that addresses the points raised during the review process.

Please submit your revised manuscript by Apr 21 2026 11:59PM. If you will need more time than this to complete your revisions, please reply to this message or contact the journal office at mentalhealth@plos.org. When you're ready to submit your revision, log on to https://www.editorialmanager.com/pmen/ and select the 'Submissions Needing Revision' folder to locate your manuscript file.

Please include the following items when submitting your revised manuscript:

- A letter that responds to each point raised by the editor and reviewer(s). You should upload this letter as a separate file labeled 'Response to Reviewers'.
- A marked-up copy of your manuscript that highlights changes made to the original version. You should upload this as a separate file labeled 'Revised Manuscript with Track Changes'.
- An unmarked version of your revised paper without tracked changes. You should upload this as a separate file labeled 'Manuscript'.

Guidelines for resubmitting your figure files are available below the reviewer comments at the end of this letter.

We look forward to receiving your revised manuscript.

Kind regards,

Gellan Karamallah Ramadan Ahmed

Academic Editor

PLOS Mental Health

**Journal Requirements:**

If the reviewer comments include a recommendation to cite specific previously published works, please review and evaluate these publications to determine whether they are relevant and should be cited. There is no requirement to cite these works unless the editor has indicated otherwise.

Please review your reference list to ensure that it is complete and correct. If you have cited papers that have been retracted, please include the rationale for doing so in the manuscript text, or remove these references and replace them with relevant current references. Any changes to the reference list should be mentioned in the rebuttal letter that accompanies your revised manuscript. If you need to cite a retracted article, indicate the article’s retracted status in the References list and also include a citation and full reference for the retraction notice.

**Additional Editor Comments (if provided):**

[Note: HTML markup is below. Please do not edit.]

Reviewers' comments:

Reviewer's Responses to Questions

**Comments to the Author**

1. If the authors have adequately addressed your comments raised in a previous round of review and you feel that this manuscript is now acceptable for publication, you may indicate that here to bypass the “Comments to the Author” section, enter your conflict of interest statement in the “Confidential to Editor” section, and submit your "Accept" recommendation.

Reviewer #2: (No Response)

**********

2. Does this manuscript meet PLOS Mental Health’s publication criteria? Is the manuscript technically sound, and do the data support the conclusions? The manuscript must describe methodologically and ethically rigorous research with conclusions that are appropriately drawn based on the data presented.

Reviewer #2: Yes

**********

3. Has the statistical analysis been performed appropriately and rigorously?

Reviewer #2: N/A

**********

4. Have the authors made all data underlying the findings in their manuscript fully available (please refer to the Data Availability Statement at the start of the manuscript PDF file)?

The PLOS Data policy requires authors to make all data underlying the findings described in their manuscript fully available without restriction, with rare exception. The data should be provided as part of the manuscript or its supporting information, or deposited to a public repository. For example, in addition to summary statistics, the data points behind means, medians and variance measures should be available. If there are restrictions on publicly sharing data—e.g. participant privacy or use of data from a third party—those must be specified.

Reviewer #2: Yes

**********

5. Is the manuscript presented in an intelligible fashion and written in standard English?

PLOS Mental Health does not copyedit accepted manuscripts, so the language in submitted articles must be clear, correct, and unambiguous. Any typographical or grammatical errors should be corrected at revision, so please note any specific errors here.

Reviewer #2: Yes

**********

6. Review Comments to the Author

Please use the space provided to explain your answers to the questions above. You may also include additional comments for the author, including concerns about dual publication, research ethics, or publication ethics. (Please upload your review as an attachment if it exceeds 20,000 characters)

**Reviewer #2:** The authors can consider refining the review question to focus more explicitly on mapping evidence on ACEs, BCEs, and PCEs among MCCYP, with child self-report treated as a secondary analytical observation rather than a core aim.

Relevant studies may have been excluded simply because they did not explicitly use ACE/BCE/PCE terminology, coming from different terminology used in other studies. The authors can consider acknowledging this limitation more explicitly and discussing how conceptual synonyms or related constructs may exist in the literature but were not captured by the search strategy.

The authors can consider discussing more explicitly how excluding grey literature may have underestimated the available evidence base, particularly in military family research where policy reports are common.

The authors can consider clarifying:

whether independent dual screening occurred

whether inter-reviewer agreement was assessed

how discrepancies were resolved.

The authors can consider clarifying:

whether CASP was used for descriptive appraisal rather than scoring, and

how study quality influenced interpretation of the findings.

The authors can consider expanding the conceptual explanation of how BCEs and PCEs differ theoretically and empirically, and why both constructs are necessary in this review.

The authors can consider further tempering conclusions, emphasising that findings primarily reflect US-based military systems and datasets.

The manuscript still includes “table ##” in the methods section. Other typographical errors also exist. So proofreading is needed.

The authors can consider softening the claim to “one of the first reviews…” or “the first review explicitly examining ACEs, BCEs, and PCEs together…”.

**********

7. PLOS authors have the option to publish the peer review history of their article (what does this mean?). If published, this will include your full peer review and any attached files.

**Do you want your identity to be public for this peer review?**  If you choose “no”, your identity will remain anonymous but your review may still be made public.

For information about this choice, including consent withdrawal, please see our Privacy Policy.

Reviewer #2: **Yes:** Peejay D. Bengwasan

**********

[NOTE: If reviewer comments were submitted as an attachment file, they will be attached to this email and accessible via the submission site. Please log into your account, locate the manuscript record, and check for the action link "View Attachments". If this link does not appear, there are no attachment files.]

-->Figure Resubmissions:

While revising your submission, we strongly recommend that you use PLOS’s NAAS tool (https://ngplosjournals.pagemajik.ai/artanalysis) to test your figure files. NAAS can convert your figure files to the TIFF file type and meet basic requirements (such as print size, resolution), or provide you with a report on issues that do not meet our requirements and that NAAS cannot fix.

After uploading your figures to PLOS’s NAAS tool - https://ngplosjournals.pagemajik.ai/artanalysis, NAAS will process the files provided and display the results in the "Uploaded Files" section of the page as the processing is complete. If the uploaded figures meet our requirements (or NAAS is able to fix the files to meet our requirements), the figure will be marked as "fixed" above. If NAAS is unable to fix the files, a red "failed" label will appear above. When NAAS has confirmed that the figure files meet our requirements, please download the file via the download option, and include these NAAS processed figure files when submitting your revised manuscript.

# Author comment: Author response to Decision Letter 5

**DOI:** 10.1371/journal.pmen.0000654.r010

20 Apr 2026

Attachment Submitted filename: Comment table ACEs V5 .docx

# Peer review: Decision Letter 5

**DOI:** 10.1371/journal.pmen.0000654.r011

**Authors:** Gellan Karamallah Ramadan Ahmed

24 Jun 2026

A Scoping Review of Adverse, Benevolent, and Positive Childhood Experiences in Military-Connected Children

PMEN-D-25-00297R5

Dear Miss Mason,

We are pleased to inform you that your manuscript 'A Scoping Review of Adverse, Benevolent, and Positive Childhood Experiences in Military-Connected Children' has been provisionally accepted for publication in PLOS Mental Health.

Before your manuscript can be formally accepted you will need to complete some formatting changes, which you will receive in a follow up email. A member of our team will be in touch with a set of requests.

Please note that your manuscript will not be scheduled for publication until you have made the required changes, so a swift response is appreciated.

IMPORTANT: The editorial review process is now complete. PLOS will only permit corrections to spelling, formatting or significant scientific errors from this point onwards. Requests for major changes, or any which affect the scientific understanding of your work, will cause delays to the publication date of your manuscript.

If your institution or institutions have a press office, please notify them about your upcoming paper to help maximize its impact. If they'll be preparing press materials, please inform our press team as soon as possible -- no later than 48 hours after receiving the formal acceptance. Your manuscript will remain under strict press embargo until 2 pm Eastern Time on the date of publication. For more information, please contact mentalhealth@plos.org.

Thank you again for supporting Open Access publishing; we are looking forward to publishing your work in PLOS Mental Health.

Best regards,

Gellan Karamallah Ramadan Ahmed

Academic Editor

PLOS Mental Health

***********************************************************

Reviewer Comments (if any, and for reference):

Reviewer's Responses to Questions

**Comments to the Author**

1. If the authors have adequately addressed your comments raised in a previous round of review and you feel that this manuscript is now acceptable for publication, you may indicate that here to bypass the “Comments to the Author” section, enter your conflict of interest statement in the “Confidential to Editor” section, and submit your "Accept" recommendation.

Reviewer #1: All comments have been addressed

**********

2. Does this manuscript meet PLOS Mental Health’s publication criteria? Is the manuscript technically sound, and do the data support the conclusions? The manuscript must describe methodologically and ethically rigorous research with conclusions that are appropriately drawn based on the data presented.

Reviewer #1: Yes

**********

3. Has the statistical analysis been performed appropriately and rigorously?

Reviewer #1: N/A

**********

4. Have the authors made all data underlying the findings in their manuscript fully available (please refer to the Data Availability Statement at the start of the manuscript PDF file)?

The PLOS Data policy requires authors to make all data underlying the findings described in their manuscript fully available without restriction, with rare exception. The data should be provided as part of the manuscript or its supporting information, or deposited to a public repository. For example, in addition to summary statistics, the data points behind means, medians and variance measures should be available. If there are restrictions on publicly sharing data—e.g. participant privacy or use of data from a third party—those must be specified.

Reviewer #1: Yes

**********

5. Is the manuscript presented in an intelligible fashion and written in standard English?

PLOS Mental Health does not copyedit accepted manuscripts, so the language in submitted articles must be clear, correct, and unambiguous. Any typographical or grammatical errors should be corrected at revision, so please note any specific errors here.

Reviewer #1: Yes

**********

6. Review Comments to the Author

Please use the space provided to explain your answers to the questions above. You may also include additional comments for the author, including concerns about dual publication, research ethics, or publication ethics. (Please upload your review as an attachment if it exceeds 20,000 characters)

Reviewer #1: I have no additional suggestions for this manuscript. My original commentary was concerning whether the lack of articles addressing the core of the research question was valid for publication. The writing and the framing is good, so if it is determined that the core concept is publishable I do not have an issue. I want to also note that the work done by the authors absolutely identifies a gap, but I was originally questioning whether that alone should be published.

**********

7. PLOS authors have the option to publish the peer review history of their article (what does this mean?). If published, this will include your full peer review and any attached files.

**Do you want your identity to be public for this peer review?**  If you choose “no”, your identity will remain anonymous but your review may still be made public.

For information about this choice, including consent withdrawal, please see our Privacy Policy.

Reviewer #1: No

**********
